# Cost-effectiveness of monotherapy and combination therapy for myopia control in Chinese children

**DOI:** 10.7189/jogh.16.04366

**Published:** 2026-10-19

**Authors:** Jinghui Wang, Xuan Li, Meinan He, Desheng Song, Bo Zhang, Bei Du, Ruihua Wei

**Affiliations:** 1 Tianjin Key Laboratory of Retinal Functions and Diseases, Tianjin Branch of National Clinical Research Center for Ocular Disease, Eye Institute and School of Optometry, Tianjin Medical University Eye Hospital, Tianjin, China; 2 TMUE Optometric Technology Co., Ltd, Tianjin, China

**Keywords:** myopia, cost-effectiveness analysis, child, network meta-analysis

## Abstract

**Background:**

Myopia poses a major public health and economic burden in China, necessitating cost-effectiveness evaluations of control strategies. We aimed to assess cost-effectiveness of single/combined myopia control in children and adolescents to optimise strategies.

**Methods:**

We conducted a network meta-analysis to evaluate the efficacy of myopia control interventions in Chinese children (aged 6–18 years). Annual changes in axial length (AL) and spherical equivalent refraction (SER) were the primary outcomes. Additionally, we used a decision-analytic Markov model with clinical and cost data from China to evaluate 16 myopia control strategies for eight-year-old children over eight years from a patient perspective, with 3% discounting. Strategies included monotherapies and combined therapies. Main outcomes were incremental cost per unit reduction in myopic shift (*i.e.* SER) or AL elongation, compared with single-vision lenses (SVLs). Markov cohort analysis simulated the proportion of individuals progressing to high myopia under various strategies. We assessed robustness through deterministic and probabilistic sensitivity analyses. We performed subgroup analyses by onset age (6, 10, and 12 years) and regional economic development.

**Results:**

For SER reduction, outdoor activity was cost-effective (incremental cost-effectiveness ratio = USD −18.29/dioptre (D)). At higher willingness-to-pay thresholds, 0.01% atropine combined with defocus incorporated multiple segment (DIMS) (USD 660.74/D) and repeated low-level red-light therapy (RLRL) (USD 895.36/D) were cost-effective. For AL reduction, outdoor activity (USD −42.68/mm) and DIMS (USD 672.73/mm) were cost-effective at lower thresholds, while RLRL (USD 2011.32/mm) was preferred at higher thresholds. Compared with monotherapy, combination interventions improved preventive efficacy against high myopia. Subgroup analyses confirmed that the cost-effectiveness ranking was preserved across onset ages and regional economic settings.

**Conclusions:**

Under limited budget conditions, outdoor activities and defocus spectacles represent advantageous cost-effective treatment options. When the budget is sufficient, RLRL may be considered a promising option pending confirmation of its long-term safety and effectiveness, and 0.01% atropine combined with DIMS is the preferred cost-effective option. Our analysis provides evidence-based guidance for clinicians and families in selecting effective myopia control strategies.

Myopia has reached epidemic levels in China, particularly among school-aged children, with a prevalence of 81.4% among urban high school students and high myopia among adolescents projected to reach 22.1% by 2050 [1]. Myopia is a major cause of blindness and visual impairment, substantially affecting quality of life and social productivity [2]. Beyond its visual consequences, myopia imposes a substantial economic burden, accounting for an estimated 0.23% of China's gross domestic product (GDP) in urban areas alone [3]. If left unchecked, progressive myopia frequently advances to high myopia, which is associated with pathological axial elongation and increased risks of sight-threatening complications including glaucoma, cataract, and retinal detachment [4–7]. Given that myopia progression is most rapid during childhood and adolescence [8], effective interventions during this critical window are essential to prevent progression to high myopia.

In response to this escalating global public health burden, numerous research efforts worldwide have been dedicated to exploring and validating diverse strategies for decelerating myopia progression [9]. Currently established interventions include defocus spectacles [10], defocus incorporated soft contact lenses (DISC) [11], atropine eye drops at different doses [12], orthokeratology (OK) [13], and the more recently recognised repeated low-level red-light therapy (RLRL) [14]. While these modalities have shown varying degrees of efficacy in controlling myopia progression in clinical studies, monotherapies often have inherent limitations or show variability in individual patient response as our understanding of myopia pathogenesis deepens and clinical demands evolve. Consequently, combination therapies, which leverage two distinct mechanisms of action – such as OK combined with low-concentration atropine – have emerged as a promising avenue. Recent studies consistently demonstrate that these combined approaches yield demonstrably superior outcomes in decelerating axial elongation and reducing refractive error progression compared to single interventions, offering a significant optimisation direction for myopia management [15–17].

The effectiveness and costs of different treatment approaches vary considerably. However, the issue of myopia is becoming increasingly severe, and the use of combination therapies to control myopia progression has become more common in clinical practice. Combination therapies have shown significant effects in delaying myopia progression [15,16,18]. However, existing studies predominantly focus on the economic evaluation of single treatments, with limited systematic analysis of the cost-effectiveness of combination therapies [19]. Additionally, there is a lack of comprehensive assessment regarding the long-term impact of different intervention strategies on the progression of high myopia. To solve these problems and provide valuable references for public health policymakers, clinicians, and families to help optimise the selection of myopia control strategies and promote rational allocation of resources, we aimed to evaluate, from the patient perspective, the cost-effectiveness of single and combined myopia control strategies for eight-year-old children with myopia in China.

## METHODS

### Search strategy and study selection

We searched PubMed, Web of Science, and the reference lists of relevant meta-analyses. We assessed study quality and conducted a network meta-analysis. We aimed to evaluate the efficacy of myopia control interventions on myopia progression in Chinese children and adolescents.

We included studies that assessed Chinese children and adolescents aged 6–18 years; publications in English; interventions aimed at retarding myopia progression in children; those that assessed annual changes in axial length (AL) and spherical equivalent refraction (SER); those with a follow-up duration of at least 1 year; and randomised controlled trials (RCTs) (**Figure 1**; Method S1 in the **Online Supplementary Document**). In addition to RCTs, we incorporated selected cohort studies into this analysis. Although the pre-specified search strategy was designed primarily to retrieve RCTs, we also identified the supplementary cohort studies through the same database searches conducted without the RCT filter, along with manual screening of the reference lists of included meta-analyses and key primary studies. We selectively incorporated these cohort studies only for intervention arms where RCT evidence was insufficient to inform the network. This decision was driven primarily by the scarcity of RCT data for certain combination therapies widely used in clinical practice in China. Excluding these interventions would not have reflected the real-world clinical landscape.

**Figure 1:**
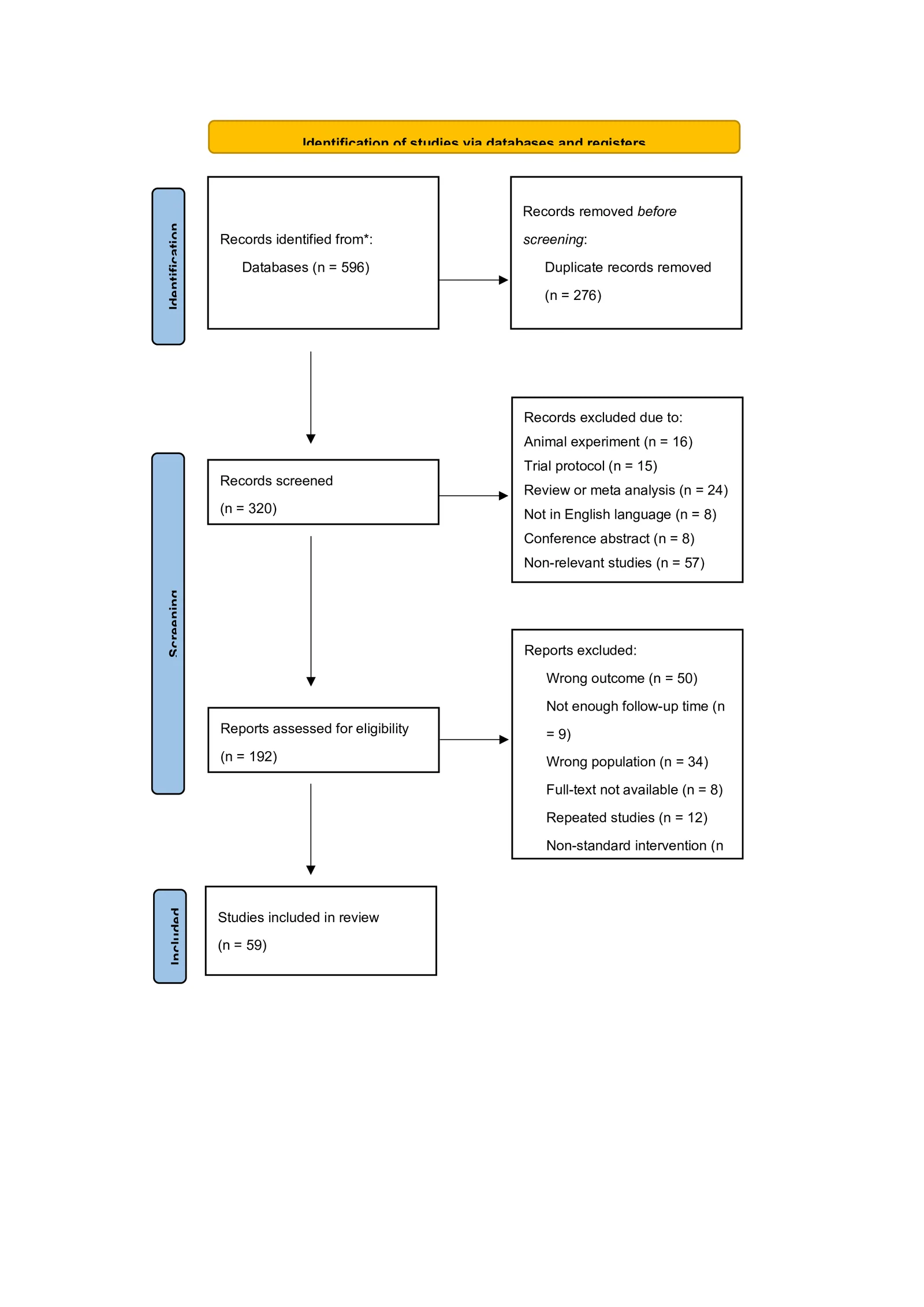
PRISMA flow diagram of study selection.

Notably, quality assessment indicated that these cohort studies provided high-quality evidence (Figure S4–7 in the **Online Supplementary Document**). To control for potential bias, we conducted a sensitivity analysis restricted to RCTs to assess the robustness of the intervention rankings. Based on the practical context of myopia control in China (*e.g.* intervention accessibility), we identified suitable strategies from the network meta-analysis results and subsequently performed a cost-effectiveness evaluation (Table S1 and S2 in the **Online Supplementary Document**).

### Cost-effectiveness analysis

#### Markov model

We constructed a comprehensive decision-analytic Markov model to simulate myopia progression over eight years in an eight-year-old myopic population and evaluate the cost-effectiveness of different interventions, because 8–16 years is the peak period of myopia progression in children and adolescents [20,21]. We used a Markov model to track annual movement between low, moderate, and high myopia over eight years. The model defined three myopia severity states: low myopia (SER ranging from −0.50 dioptre (D) to −2.99 D), moderate myopia (SER ranging from −3.00 D to −5.99 D), and high myopia (SER≤−6.00 D). Individuals could transition annually to more severe states, and these transitions were irreversible (Figure S1 in the **Online Supplementary Document**). We determined transition probabilities using a state transition probability matrix with a one-year cycle. Because the Markov model uses discrete one-year cycles, we applied a half-cycle correction to all costs and effectiveness outcomes to avoid overestimating the costs and health outcomes associated with each state. The model incorporated the effects of multiple interventions on transition probabilities, including single-vision lenses (SVLs), 0.01% atropine eye drops, specially designed spectacles (*i.e.* defocus incorporated multiple segment (DIMS), highly aspherical lenslet (HAL), cylindrical annular refractive element, diversified segmental defocus optimisation (DSDO), and progressive addition lens), DISC, OK, RLRL, outdoor activity, and various combined therapies (0.01% atropine + DIMS, 0.01% atropine + HAL, 0.01% atropine + DSDO, 0.01% atropine + OK). Except for OK, DISC, and specially designed spectacles, we assumed the other interventions were used with additional SVLs, and we set the SVL group as the control group.

Based on a large cohort study, we set the initial proportion of patients with high myopia at 4.24% [22]. Given the eligibility criteria for OK, the OK and 0.01% atropine combined cohorts initially excluded participants with high myopia. Individuals who developed high myopia during follow-up retained their assigned treatment. To assess cost-effectiveness relative to SVL, the model incorporated the medical costs and effectiveness outcomes of each intervention (*e.g.* improvements in SER and AL) and calculated the incremental cost-effectiveness ratio (ICER) to assess resource allocation efficiency (Figure S2 in the **Online Supplementary Document**).

### Parameter settings and sources

#### State transition probabilities

The state transition probabilities of myopia progression describe the transitions among low, moderate, and high myopia states. We derived the initial myopia-state probability distribution from the distribution of low, moderate, and high myopia among students in grades 1–3 in Hubei Province, China [22]. Following Welton and Ades [23], we extracted SER progression data from RCTs on different myopia control strategies and calculated the myopia state transition and stability probabilities for each state under different strategies in the model (Methods S2, Table S3 in the **Online Supplementary Document**). To account for age effects on the effectiveness of myopia control methods, we made the transition matrices time-heterogeneous, allowing the probability of transitioning to more severe myopia states to decrease with age. Based on the natural history of myopia progression in children aged 7–17 years reported by Moore *et al.* [24], we set the state transition probabilities after age eight to decrease by 5% annually.

#### Cost parameters

The cost parameters included both direct and indirect costs. We based direct costs (examination and treatment) on pricing data from hospitals or optometry centres across representative regions of China, including eastern, western, southern, northern, and central areas, reflecting regional market averages (Figure S3 in the **Online Supplementary Document**). We set the cost of outdoor activity as the cost of wearing SVLs. Indirect costs include productivity loss during treatment or follow-up visits. Because this model evaluated long-term outcomes, we applied a discount rate to convert future health benefits into costs. In this study, we discounted all future costs and benefits at 3% [25]. We estimated productivity loss based on the national average wage level in China released by the National Bureau of Statistics in 2024 [26], assuming 2 hours spent on medical visits. Costs were converted to USD (USD 1 = CNY 7.14) [27]. Willingness-to-pay (WTP) was defined as the maximum amount willing to pay for one additional unit of health benefit. Clinical outcomes served as the measure of effectiveness in this study. Based on previous literature and clinical expert consensus [19], we set the WTP threshold range at USD 0–10,000 per unit of health gain. The final cost parameters included in the analysis are shown in **Table 1**.

**Table 1:** Average annual costs and improvements in SER and AL by intervention*

	Average annual spending (USD)	Annual improvement in SER (D)	Annual improvement in AL (mm)
**SVL†**	156.99	−0.81	−0.36
**Outdoor**	156.99	0.1	0.03
**DIMS**	493.36	0.23	0.14
**HAL**	496.01	0.26	0.13
**DSDO**	571.46	0.24	0.14
**CARE**	483.77	0.21	0.10
**PDMSL**	315.57	−0.34	−0.19
**PAL**	350.03	0.10	0.01
**0.01% Atr**	619.52	0.22	0.09
**DISC**	1,566.61	0.45	0.16
**OK**	1,090.49		0.15
**RLRL**	1,544.81	0.74	0.33
**0.01% Atr + DIMS**	1,082.03	0.58	0.24
**0.01% Atr + HAL**	1,084.69	0.27	0.17
**0.01% Atr + DSDO**	1,160.14	0.32	0.14
**0.01% Atr + OK**	1,673.42		0.15

AL – axial length, Atr – atropine, CARE – cylindrical annular refractive element, D – dioptre, DIMS – defocus incorporated multiple segments, DISC – defocus incorporated soft contact, DSDO – diversified segmental defocus optimisation, HAL – highly aspherical lenslet, OK – orthokeratology, PAL – progressive addition lens, PDMSL – peripheral defocus modifying spectacle lens, RLRL – repeated low-level red-light therapy, SER – spherical equivalent refraction, SVL – single-vision lens

*Discount rate was at 3.0% [25] and hourly lost wages at USD 8.33 [26].

†Values for annual improvement in SER and AL extracted from Bao *et al.* (2021) [40].

#### Effectiveness parameters

We used improvements in SER progression and AL elongation relative to the SVL comparator, as they are commonly used clinical indicators for assessing myopia progression. By comparing intervention costs with SER or AL improvements, we calculated the ICER for each strategy to evaluate the cost-effectiveness of different interventions. We simulated health outcomes and state transitions over eight years based on published literature.

#### Subgroup analyses

To investigate the influence of intervention initiation age and regional economic development on the cost-effectiveness of myopia control strategies, we performed additional subgroup analyses stratified by age and by cost setting. For the age-stratified analysis, we estimated cost-effectiveness for cohorts initiating intervention at ages 6, 10, and 12. For the cost-stratified analysis, we classified intervention costs as prevailing in economically developed regions or less developed regions, based on each area's economic status, and re-ran the cost-effectiveness model separately for each cost setting to assess strategy performance under different economic contexts.

Finally, to compare the preventive effects of various myopia control interventions against the development of high myopia, we performed a cohort simulation analysis. We assumed multiple cohorts of eight-year-old children, all with low myopia at baseline (*i.e.* a baseline prevalence of low myopia of 100%), and compared the prevalence of high myopia across cohorts after eight years of receiving each intervention.

### Sensitivity analysis

Given the inevitable uncertainty in model parameters, we conducted one-way deterministic sensitivity analyses. Specifically, we assessed the impact of parameter variation using a ±20% range for direct costs, 95% confidence intervals (CIs) for health effects, and reasonable variation ranges for age at intervention initiation, cohort time horizon, productivity loss, and the discount rate. We set the minimum cost of outdoor activities to 0, as outdoor sports may have no associated costs. We also performed a probabilistic sensitivity analysis. We assumed cost parameters follow a gamma distribution and effectiveness parameters follow a normal distribution. Using Monte Carlo simulation, we evaluated the probability of cost-effectiveness for each strategy under different WTP thresholds, with costs and effects following the specified probability distributions and 1000 iterations. All parameter variations used in the sensitivity analyses were supported by the literature or fell within reasonable ranges, ensuring the robustness and reliability of our results.

#### Software

We conducted the study quality assessment and network meta-analysis using Review Manager, version 5.4 (The Cochrane Collaboration, London, UK), CINeMA [28], and *R* software, version 4.4.2 (R Core Team, Vienna, Austria). For the Markov model, we used TreeAge Pro, version 2022 R1.2 (Williamstown, Massachusetts, USA).

## RESULTS

### Network meta-analysis

We included 59 studies in the network meta-analysis (Table S1–2 in the **Online Supplementary Document**). Because of the nature of certain interventions (*e.g.* RLRL, OK), double-blinding of participants was not feasible in some RCTs, a recognised methodological limitation in this field. In addition, the main risk to the literature quality of cohort studies stemmed from the fact that the exposed population consisted of volunteer hospital-based cohorts rather than community-based randomly selected individuals. However, this is generally considered acceptable in myopia control research. Nevertheless, quality assessment indicated that the overall quality of the included studies was acceptable (Figure S4–7 in the **Online Supplementary Document**), ensuring high reliability. Results from the network meta-analysis (Figure S8 and S9 in the **Online Supplementary Document**) demonstrated that interventions such as 0.01% atropine, DIMS, DISC, and RLRL significantly mitigated the myopic shift in SER compared with controls. Furthermore, 0.01% atropine, DIMS, DISC, DSDO, HAL, OK, and RLRL showed significant efficacy in retarding AL elongation. Sensitivity analyses showed that the intervention rankings remained largely unchanged regardless of whether cohort studies were included (Figure S10 in the **Online Supplementary Document**), indicating the robustness of our core results.

Additionally, using the CINeMA framework, we assessed confidence in the network meta-analysis estimates across six domains (within-study bias, reporting bias, indirectness, imprecision, heterogeneity, and incoherence). We rated overall confidence as moderate for all key comparisons (based on 51 RCTs; 1–12 direct studies per comparison), with some concerns about heterogeneity and imprecision in some indirect comparisons (Table S4 in the **Online Supplementary Document**). No comparisons were rated as low or very low confidence.

The network meta-analysis (Figure S8 in the **Online Supplementary Document**) demonstrated that RLRL achieved the largest treatment effect among monotherapies, with a mean SER reduction of 0.74 D (95% CI = 0.62–0.85) and an AL elongation reduction of 0.33 mm (95% CI = 0.29–0.38) relative to SVL. Among combination therapies, 0.01% atropine combined with DIMS showed the greatest effect for both SER (0.59 D; 95% CI = 0.18–0.99) and AL (0.24 mm; 95% CI = 0.16–0.31). The RCT-only sensitivity analysis (Figure S10 in the **Online Supplementary Document**) confirmed the robustness of these estimates: RLRL remained the most effective monotherapy for SER (0.74 D; 95% CI = 0.61–0.86) and AL (0.33 mm; 95% CI = 0.29–0.38), and 0.01% atropine combined with DIMS showed a consistent AL effect (0.20 mm, 95% CI = 0.05–0.34). The SER estimate for 0.01% atropine combined with DIMS was derived exclusively from cohort studies, as no RCT evidence was available for this combination.

Specifically, the SER effect estimates for 0.01% atropine combined with DIMS and 0.01% atropine combined with HAL were derived exclusively from cohort studies, as no RCT evidence was available for these specific combinations; in the RCT-only sensitivity analysis, these two combinations could not be estimated. For the AL outcome, 0.01% atropine combined with HAL likewise relied entirely on cohort evidence. For all other interventions, the mixed and RCT-only NMA estimates were conducted using the same statistical method (netmeta, random-effects model) to ensure methodological consistency; the estimates were nearly identical for the vast majority of interventions (difference <10%), confirming that the inclusion of cohort studies did not materially alter the effect sizes. The only intervention showing a notable difference was HAL for SER (53.8%) and AL (38.5%), attributable to the removal of one multi-arm cohort study that provided both direct HAL evidence and indirect network connectivity; however, the 95% CIs still overlapped between the two analyses, indicating that the difference was within the range of statistical uncertainty (Table S5 in the **Online Supplementary Document**).

### Cost-effectiveness analysis and Markov cohort simulation

Among monotherapies, outdoor activity had the lowest total cost (USD 1118.58), whereas DISC had the highest total cost (USD 11,280.65) (**Figure 2**, Panels A and B; **Table 2**). Among the combined therapies, the lowest eight-year cost was for 0.01% atropine combined with DIMS (USD 7828.08), while the highest was for 0.01% atropine combined with DSDO (USD 8384.57). Regarding improvements in SER and AL across interventions, RLRL achieved the best effect among monotherapies, and 0.01% atropine combined with DIMS achieved the best effect among the combined therapies.

**Figure 2:**
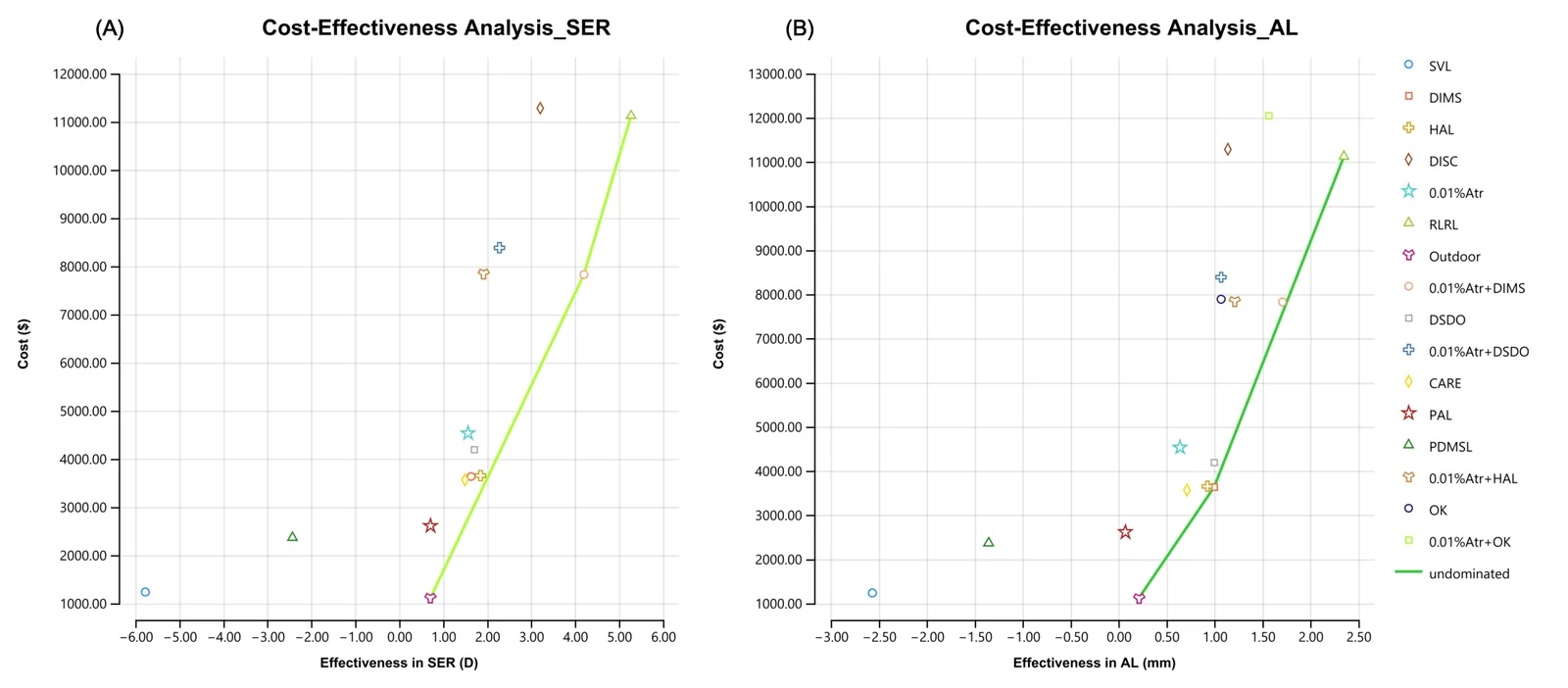
Cost-effectiveness analysis results for SER and AL. **Panel A.** SER. **Panel B.** AL. AL – axial length, SER – spherical equivalent refraction. 0.01%Atr – 0.01% atropine, CARE – cylindrical annular refractive element, DIMS – defocus incorporated multiple segment, DISC – defocus incorporated soft contact, DSDO – diversified segmental defocus optimization, HAL – highly aspherical lenslets, OK – orthokeratology, PAL – progressive addition lens, PDMSL – peripheral defocus modifying spectacle lens, RLRL – repeated low-level red-light therapy; SVL – single-vision lens. Undominated refers to strategies that are not dominated by any other strategy in the cost-effectiveness plane.

**Table 2:** Cost-effectiveness analysis of therapies based on SER and AL improvement

	SER					AL				
	**Cost**	**Incr cost**	**Eff**	**Incr eff**	**ICER**	**Cost**	**Incr cost**	**Eff**	**Incr eff**	**ICER**
**SVL**	1,237.17		−5.77			1,237.17		−2.56		
**Outdoor**	1,118.58	−118.59	0.71	6.48	−18.29	1,118.58	−118.59	0.21	2.78	−42.68
**PAL**	2,612.55	1,375.37	0.71	6.48	212.13	2,612.55	1,375.37	0.07	2.64	521.72
**HAL**	3,652.64	2,415.47	1.85	7.62	316.84	3,652.64	2,415.47	0.93	3.49	691.86
**CARE**	3,565.44	2,328.26	1.50	7.27	320.37	3,565.44	2,328.26	0.71	3.28	710.38
**DIMS**	3,633.76	2,396.59	1.64	7.41	323.43	3,633.76	2,396.59	1.00	3.56	672.73
**PDMSL**	2,367.00	1,129.82	−2.42	3.35	337.39	2,367.00	1,129.82	−1.35	1.21	932.77
**DSDO**	4,190.25	2,953.07	1.71	7.48	394.73	4,190.25	2,953.07	1.00	3.56	828.93
**0.01%Atr**	4,532.65	3,295.48	1.57	7.34	449.05	4,532.65	3,295.48	0.64	3.21	1,027.83
**0.01%Atr + DIMS**	7,828.08	6,590.90	4.20	9.97	660.74	7,828.08	6,590.90	1.71	4.27	1,541.73
**0.01%Atr + HAL**	7,846.97	6,609.79	1.92	7.69	858.97	7,846.97	6,609.79	1.21	3.78	1,750.36
**0.01%Atr + DSDO**	8,384.57	7,147.40	2.28	8.05	887.74	8,384.57	7,147.40	1.07	3.63	1,966.95
**RLRL**	11,125.33	9,888.15	5.27	11.04	895.36	11,125.33	9,888.15	2.35	4.92	2,011.32
**DISC**	11,280.65	10,043.48	3.21	8.98	1,118.74	11,280.65	10,043.48	1.14	3.70	2,710.79
**OK**						7,888.33	6,651.15	1.07	3.63	1,830.38
**0.01%Atr + OK**						12,041.69	10,804.51	1.57	4.13	2,614.52

AL – axial length, Atr – atropine, CARE – cylindrical annular refractive element, DIMS – defocus incorporated multiple segment, DISC – defocus incorporated soft contact, DSDO – diversified segmental defocus optimisation, Eff – effectiveness, HAL – highly aspherical lenslet, ICER – incremental cost-effectiveness ratio, Incr – incremental, OK – orthokeratology, PAL – progressive addition lens, PDMSL – peripheral defocus modifying spectacle lens, RLRL – repeated low-level red-light therapy, SER – spherical equivalent refraction, SVL – single-vision lens

In the cost-effectiveness analysis based on SER improvement, outdoor activity, 0.01% atropine combined with DIMS, and RLRL were the dominant strategies. In the cost-effectiveness analysis based on AL improvement, outdoor activity, DIMS, and RLRL were cost-effective. Compared with SVL, outdoor activity yielded cost savings of USD 16.66/SER reduction and USD 36.99/AL reduction. The ICERs of RLRL for reducing SER progression was USD 895.36/D and USD 2011.32/mm for reducing AL elongation. The combination of 0.01% atropine with DIMS would delay one unit of SER progression at a cost of USD 660.74. The DIMS would achieve a delay of one unit of AL elongation at a cost of USD 672.73. The ICERs of these interventions were all below the WTP threshold.

The eight-year cohort analysis revealed that combination therapies were superior to monotherapies in delaying the onset of high myopia. Under the SVL comparator, the eight-year cumulative incidence of high myopia was 75.00%. Relative to the respective monotherapy, the addition of 0.01% atropine reduced the eight-year cumulative incidence by 0.58% when combined with DIMS and by 27.32% when combined with DSDO (Table S6 in the **Online Supplementary Document**). These findings suggest that combination therapies may serve as the preferred strategy for preventing high myopia, offering substantial long-term benefits.

### Deterministic sensitivity analysis and probabilistic sensitivity analysis

Deterministic sensitivity analysis (Figures S11–14 in the **Online Supplementary Document**) indicated that the costs and efficacy of the interventions were the key drivers of the cost-effectiveness rankings. In contrast, the discount rate, starting age, and productivity loss exerted minor influence.

When WTP ranged from USD 0 to 1000/SER reduction, outdoor activity was the optimal myopia control strategy, with a cost-effectiveness acceptability probability of 92.9% (**Figure 3**, Panel A). When WTP increased to USD 1500/SER reduction, outdoor activity had a cost-effectiveness acceptability probability of 24.8%. Beyond this threshold, outdoor activity might no longer be the preferred strategy, and 0.01% atropine combined with DIMS became competitive strategies, with cost-effectiveness acceptability probabilities of 31.7%. RLRL became the preferred myopia control strategy when the WTP exceeded USD 4000/SER reduction, with the highest cost-effectiveness acceptability probability reaching 62.4%. The 0.01% atropine combined with DIMS ranked second; although its cost-effectiveness acceptability probability gradually declined within this WTP range, it generally remained above 32%.

**Figure 3:**
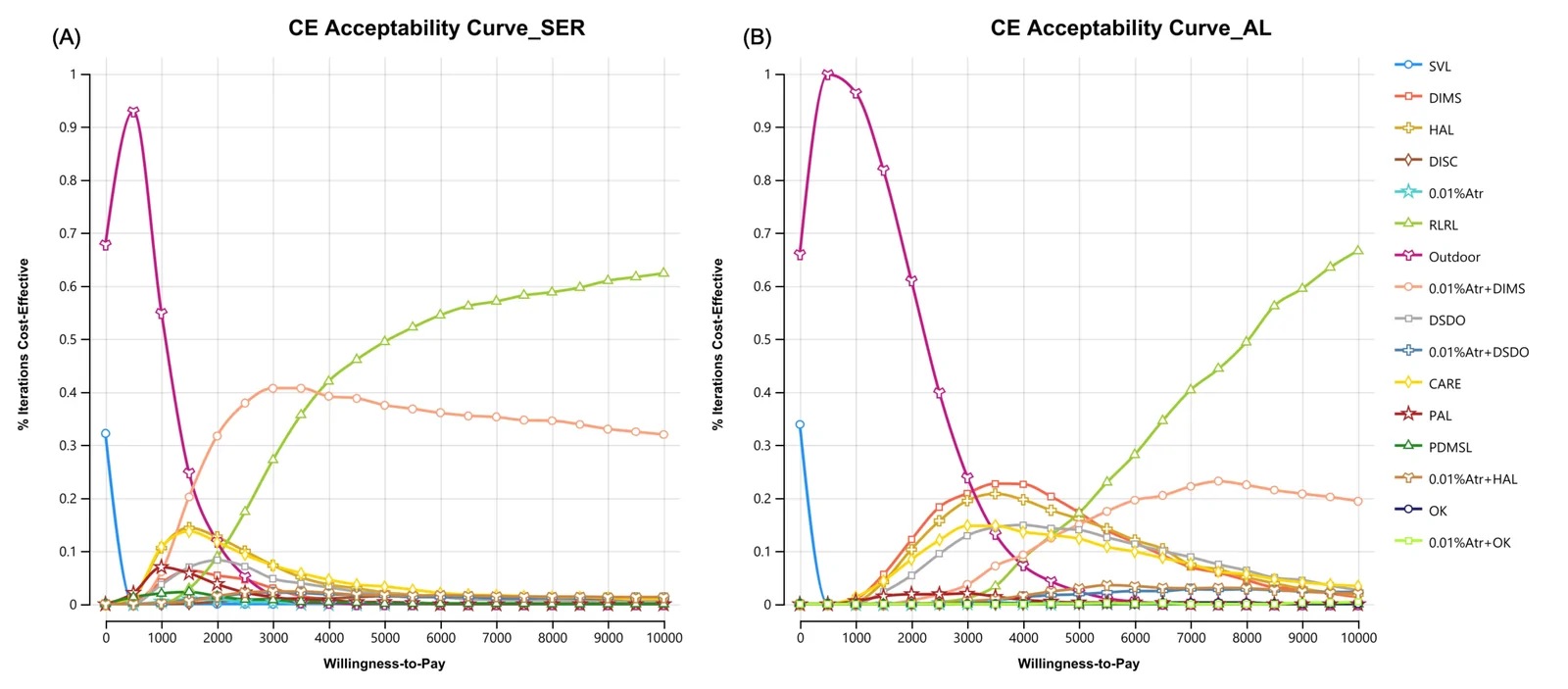
Probabilistic sensitivity analysis results for SER and AL. **Panel A.** SER. **Panel B.** AL. AL – axial length, SER – spherical equivalent refraction. 0.01%Atr – 0.01% atropine, CARE – cylindrical annular refractive element, DIMS – defocus incorporated multiple segment, DISC – defocus incorporated soft contact, DSDO – diversified segmental defocus optimization, HAL – highly aspherical lenslets, OK – orthokeratology, PAL – progressive addition lens, PDMSL – peripheral defocus modifying spectacle lens, RLRL – repeated low-level red-light therapy; SVL – single-vision lens.

When WTP ranged from USD 0 to 500/AL reduction, outdoor activity was cost-effective at 100% (**Figure 3**, Panel B). When the WTP exceeded USD 3500/AL reduction, DIMS (22.1%) and HAL (17.2%) replaced outdoor activity as the cost-effective strategy. RLRL was the best myopia control intervention when the WTP reached USD 5500/AL reduction or higher, with a cost-effectiveness acceptability probability of up to 67.9%. The second-best strategy was 0.01% atropine combined with DIMS, with a cost-effectiveness acceptability probability of 21.0%.

Overall, outdoor activity was cost-effective at lower WTP thresholds. As the WTP increased, DIMS or DIMS combined with 0.01% atropine gradually became the optimal strategies. At higher WTP levels, RLRL was the most advantageous option.

### Subgroup analyses

We performed age-stratified cost-effectiveness analyses for cohorts initiating intervention at ages 6, 10, and 12 (Tables S8–10 in the **Online Supplementary Document**). Across all onset ages, the cost-effectiveness ranking of interventions remained the same. For SER reduction, outdoor activity remained cost-saving at ages 6 (ICER = USD −18.96), 10 (ICER = USD −17.82), and 12 (ICER = USD −18.76). For AL reduction, DIMS showed stable ICERs at ages 6 (ICER = USD 670.64), 10 (ICER = USD 666.34), and 12 (ICER = USD 662.31). Notably, RLRL remained the highest cost but most effective monotherapy for AL reduction across ages 6 (ICER = USD 2025.57), 10 (ICER = USD 2025.17), and 12 (ICER = USD 2027.64). The ICERs for all key interventions varied by less than 1% across onset ages.

Cost-stratified analysis by regional economic development (Tables S7, S11, and S12 in the **Online Supplementary Document**) revealed that ICERs varied substantially across regions. In less economically developed regions, the ICER for RLRL was lower for both AL (USD 1664.42) and SER (USD 740.94) than the national average for AL (USD 2011.32) and SER (USD 895.36). In contrast, the ICER for RLRL was higher in highly developed regions for both AL (USD 2376.12) and SER (USD 1057.76). For DIMS, the AL ICER was USD 704.48/mm in less developed and USD 633.98/mm in highly developed regions. Outdoor activity remained cost-saving across all regional settings for SER (ICER = USD −18.29/D). Despite absolute cost differences, the relative ranking of interventions was preserved across all regional settings.

## DISCUSSION

To our knowledge, this is the first cost-effectiveness analysis of myopia control strategies, including combination therapies, based on data from mainland China. This study covered 16 myopia control strategies, including 12 monotherapies and 4 combination therapies. When WTP was under USD 1500/D reduction or USD 3000/mm reduction, outdoor activity was the cost-effective option because of its low cost. Above this threshold, DIMS or a combination of 0.01% atropine and RLRL was more cost-effective.

Although the probabilities of cost-effectiveness of different interventions vary under different WTP thresholds, according to the World Health Organization definition, when the cost of an intervention is lower than the per-capita GDP, the intervention can be considered highly cost-effective [29]. In this study, the ICERs for all interventions were below the per-capita GDP threshold (USD 13,362.69). Therefore, compared with SVL, all strategies can be considered cost-effective. This per-capita GDP benchmark is a heuristic threshold based on economic output rather than a utility-based threshold expressed in quality-adjusted life-years (QALY). Because no utility weights were assigned to the myopia health states and clinical outcomes (SER and AL), and quality-adjusted life years served as the measure of effectiveness, interpret the cost-effectiveness conclusions with caution; they are not directly comparable to QALY-based health technology assessments from other healthcare systems.

To contextualise the practical significance of the cost-effectiveness acceptability curve threshold crossovers, each critical WTP threshold can be expressed as a fraction of China’s per-capita GDP (USD 13,362.69 ≈ CNY 95,410). The threshold at which outdoor activity ceases to dominate (≈ USD 1500/D of SER reduction) corresponds to only 11.2% of per-capita GDP, or about 179.5 hours of labour (at the national average hourly wage of CNY 59.67), indicating that this strategy remains affordable for most households. In contrast, the threshold at which RLRL becomes the preferred strategy (≈ USD 4000/D) corresponds to 29.9% of per-capita GDP, or about 478.6 labour hours. At present, myopia control interventions are not covered by basic medical insurance in China, so families bear these costs entirely out of pocket. Consequently, while strategies whose ICERs fall well below the per-capita GDP threshold (*e.g.* outdoor activity, DIMS) represent a modest financial burden relative to typical household income, higher-cost interventions such as RLRL may be prohibitive for lower-income families without additional financial support. These figures underscore the equity advantage of low-cost interventions such as outdoor activity, and highlight the need for targeted financial assistance programmes, such as subsidised access or tiered pricing schemes, to ensure that higher-cost strategies are accessible to families across all income levels.

Because of the minimal direct financial outlay, outdoor activities are widely recognised as a foundational, universally applicable option in myopia control strategies. However, substantial evidence indicates that merely increasing outdoor activity time offers limited efficacy in slowing myopia progression, particularly in children and adolescents with established myopia and those in rapid progression phases [30]. Consequently, integrating outdoor activities with other myopia control interventions is widely advocated as a more effective, comprehensive strategy [30,31]. Extensive evidence robustly supports the efficacy of specialised optical spectacle lenses (*e.g.* DIMS, DSDO, HAL) designed on the principle of myopia defocus [10,32–34]. In China, these defocus-incorporated spectacle lenses have become one of the most widely adopted active interventions in clinical practice [35], owing to their non-invasive nature, conventional wearing method, high patient acceptance and adherence, and applicability across a broad spectrum of refractive states and age groups. The results of the current study indicate that the ICER associated with using defocus-incorporated spectacle lenses is higher than that for outdoor activities. This trend aligns with findings from a health economic study conducted in Hong Kong, China [19]. A further comparison of cost structures revealed that the costs estimated in the current study, based on data from mainland China, were lower than those reported for Hong Kong. This discrepancy is primarily attributed to differences in healthcare service systems and pricing standards between the two regions.

Clinically, an annual AL increase of less than 0.2 mm and a myopic shift of no more than 0.50 D in children aged 6–12 years are considered ideal myopia control outcomes [36,37]. However, with the onset age of myopia gradually becoming younger and the progression rate continuously accelerating, a single control strategy often fails to achieve an ideal effect [38]. Combined approaches are considered effective for improving myopia control outcomes [35]. Therefore, evaluating the cost-effectiveness of combined myopia control therapies is important. Among the combined therapies of optical lenses and medications in this study, 0.01% atropine combined with DIMS therapy was the most cost-effective option. Although this strategy cost more than monotherapies (USD 660.74/SER reduction and USD 1541.73/AL reduction), it could reduce the proportion of high myopia by 48% after eight years of age. At a WTP threshold of USD 1800, this therapy was considered a highly desirable option. Among the monotherapies, RLRL performed best. If the budget allows, RLRL is preferable to 0.01% atropine combined with DIMS and is the optimal choice. Previous studies have confirmed the strong myopia-controlling effect of RLRL [14,39]. For example, Xu *et al.* found that within one year, 53.3% of patients had an AL shortening of >0.05 mm [39]. Robust long-term safety and effectiveness data for RLRL remain scarce, as most available trials have relatively short follow-up durations. Further well-designed longitudinal studies with extended follow-up are needed to confirm RLRL's sustained efficacy and safety profile before it can be firmly endorsed as a standard myopia control intervention.

In contrast to low-to-moderate myopia, high myopia is characterised by progressive excessive axial elongation and sustained deepening of refractive error. These structural alterations significantly increase the risk of pathological damage to the posterior segment of the eye, including macular degeneration, retinal detachment, and optic nerve damage [2,36]. These severe complications impose a substantial health burden on patients, leading to permanent vision impairment and even blindness. Moreover, their complex, protracted, and costly treatments place continuous, heavy pressure on both social healthcare systems and family economies [37,38]. In the eight-year simulated cohort, the combination of 0.01% atropine and defocus spectacles showed significantly greater efficacy in delaying high-myopia progression than monotherapies. This significant risk reduction, combined with the inherent advantages of the intervention – namely its broad applicability and ease of implementation – positions the combined therapy of defocus spectacles and atropine not only as an effective tool for slowing myopia progression but also as a pivotal public health strategy for preventing high myopia and its associated irreversible ocular morbidities.

The age-stratified subgroup analysis demonstrated that the cost-effectiveness ranking of interventions is age-invariant, with ICERs varying by less than 1% across onset ages. This finding is clinically reassuring; while younger children experience faster myopia progression and thus greater absolute benefit from intervention, each strategy's relative economic efficiency remains constant. Clinicians and policymakers can therefore apply the same tiered cost-effectiveness framework – outdoor activity at low WTP, DIMS at moderate WTP, and RLRL at high WTP – regardless of the specific age at which myopia is first detected, without needing to prioritise different strategies for different age subgroups.

The regional subgroup analysis showed that while absolute ICERs varied across regions with different levels of economic development, the intervention ranking remained consistent. Notably, myopia control interventions are not currently covered by basic medical insurance in China; the higher absolute out-of-pocket costs in developed regions may paradoxically improve relative access, as families in these areas typically have higher disposable income, whereas the lower costs in less developed regions, while more affordable in absolute terms, may still represent a substantial burden relative to local income. These findings suggest that regional cost variation should be considered when designing equitable myopia control policies, and that targeted financial assistance programmes, such as subsidised access or tiered pricing adapted to regional economic conditions, would be important measures to ensure equitable access across regions with differing levels of economic development.

This study has several limitations. First, direct and indirect costs may vary across regions, which may affect the generalisability of the conclusions. To mitigate this, we collected cost data from optometry centres and ophthalmic hospitals across different regions of China. Additionally, deterministic sensitivity analysis showed that productivity loss had minimal impact on the results, reinforcing the robustness of our conclusions. Second, this study did not account for differences in myopia control effectiveness and their impact on future economic burden. For example, high myopia may impose a heavy socioeconomic burden. To address this limitation, we indirectly reflected potential economic losses by predicting the impact of different myopia control effects on the prevalence of high myopia after eight years. Third, we assumed complete treatment adherence and did not account for rebound effects after discontinuation, mainly because evidence on this issue remains scarce. Therefore, further research and meta-analyses are urgently needed to strengthen the evidence base in this field. Fourth, the Markov model simplifies the disease pathway by modelling only transitions among low, moderate, and high myopia and does not incorporate downstream vision-threatening complications of high myopia (*e.g.* glaucoma, cataract, and retinal detachment) or vision-related quality of life; consequently, the analysis may underestimate the long-term health benefits of delaying high-myopia onset. Also, the eight-year time horizon is insufficient to capture the lifelong consequences of myopia progression, as high-myopia complications typically manifest in adulthood. Fifth, because the literature search was restricted to English-language publications, we cannot exclude potential language bias, and relevant studies published in other languages may have been missed. Sixth, the evidence base for RLRL is still emerging, and further investigation is needed to determine the long-term safety and effectiveness of this intervention. Finally, because clinical outcomes (SER and AL) rather than QALY-based measures were used as the effectiveness metric, the cost-effectiveness results are not directly comparable with QALY-based health technology assessments, and the GDP-based threshold should be regarded as a heuristic benchmark rather than a formal utility-based WTP criterion.

## CONCLUSIONS

From a health economic perspective, outdoor activities are the primary, preferred interventions for myopia control because of their outstanding cost-effectiveness. In resource-limited settings, defocus spectacles offer demonstrated myopia-slowing efficacy with a relatively modest financial outlay. Conversely, for families with sufficient resources, combination therapies – such as 0.01% atropine eye drops combined with DIMS – and RLRL may be considered promising options for improving myopia control outcomes, pending confirmation of their long-term safety and effectiveness, albeit at a higher direct medical cost. Therefore, when superior clinical outcomes and financial affordability are not constraints, combination strategies represent the optimal choice.

## Additional material


Online Supplementary Document

## Data Availability

**Data availability:** The original contributions presented in the study are included in the article/Online Supplementary Document; further inquiries can be directed to the corresponding author.
