# Supplementary material for: Cost-effectiveness of monotherapy and combination therapy for myopia control in Chinese children
Source: J Glob Health. 2026 Oct 19;16:04366. doi: 10.7189/jogh.16.04366 (PMC13679346; doi:10.7189/jogh.16.04366)
Supplement: Supplementary file 1 [file jogh-16-04366-s001.pdf]

Supplement to: Wang JH, Li X, He MN, Song DS, Zhang B, Du B, Wei RH. Cost-effectiveness of monotherapy and combination therapy for myopia control in Chinese children. J Glob Health. 2026;16:04366.

Cost-Effectiveness of Monotherapy and Combination Therapy for Myopia Control in Chinese Children

Contents

Methods S1: Search strategy for meta-analysis. 3

Methods S2: Construction of the State Transition Probability Matrix. 4

Table S1. Characteristics of included studies in the network meta-analysis (SER). 5

Table S2. Characteristics of included studies in the network meta-analysis (AL). 8

Table S3. Sources of literature on transition probabilities. 12

Table S4. CINeMA Confidence Assessment for Network Meta-Analysis Estimates. 15

Table S5. Mixed NMA vs RCT-only NMA: Comparison of Effect Estimates (vs SVL control). 17

Table S6. Simulation results for the proportion of high myopia in an 8-year-old population with newly diagnosed myopia. 19

Table S7. Average annual costs ($) of myopia control interventions by regional economic development level. 20

Table S8. Cost-effectiveness results of myopia control interventions for cohorts with onset at age 6. 21

Table S9. Cost-effectiveness results of myopia control interventions for cohorts with onset at age 10. 22

Table S10. Cost-effectiveness results of myopia control interventions for cohorts with onset at age 12. 23

Table S11. Cost-effectiveness results of myopia control interventions in less economically developed regions. 24

Table S12. Cost-effectiveness results of myopia control interventions in highly developed regions. 25

Figure S1. Markov chain of myopia state transitions. 26

Figure S2. Design and Analytical Framework for Cost-Effectiveness Analysis of Childhood Myopia Control Strategies Based on a Markov Decision Model. 27

Figure S3. Distribution of sources for cost data in China**.** 28

Figure S4. Risk of Bias Assessment (RCT). 29

Figure S5. Summary of Risk of Bias in RCTs. 30

Figure S6. Risk of Bias Assessment (Cohort). 31

Figure S7. Summary of Risk of Bias in Cohorts. 32

Figure S8. Forest plot for the control effects on SER and AL. Panel A. SER. Panel B. AL. 33

Figure S9. Network of myopia control interventions. 34

Fi**gure S10**. Forest plot for the control effects on SER and AL (including RCTs only). Panel A. SER. Panel B. AL. 35

Figure S11. Tornado Diagram of Sensitivity Analysis on the Effect of SER. 36

Figure S12. Tornado Diagram of Sensitivity Analysis on the Effect of SER. 37

Figure S13. Tornado Diagram of Sensitivity Analysis on the Effect of AL. 38

Figure S14. Tornado Diagram of Sensitivity Analysis on the Effect of AL. 39

Methods S1: Search strategy for meta-analysis.

PubMed:

("Myopia"[Mesh] OR myopia[Title/Abstract] OR "near sighted*"[Title/Abstract] OR "short sight*"[Title/Abstract] OR "refractive error*"[Title/Abstract])

AND

("Myopia/prevention and control"[Mesh] OR prevention[Title/Abstract] OR control[Title/Abstract] OR management[Title/Abstract] OR intervention*[Title/Abstract] OR treatment[Title/Abstract] OR therapy[Title/Abstract] OR orthokeratology[Title/Abstract] OR "ortho-k"[Title/Abstract] OR atropine[Title/Abstract] OR spectacle*[Title/Abstract] OR "contact lens*"[Title/Abstract] OR "red light"[Title/Abstract] OR RLRL[Title/Abstract] OR "low-level laser"[Title/Abstract] OR multifocal[Title/Abstract] OR "peripheral defocus"[Title/Abstract])

AND

("China"[Mesh] OR China[Title/Abstract] OR Chinese[Title/Abstract] OR "Hong Kong"[Title/Abstract] OR "Macau"[Title/Abstract] OR "Taiwan"[Title/Abstract] OR PRC[Title/Abstract] OR "People's Republic of China"[Title/Abstract])

AND

("Randomized Controlled Trial"[Publication Type] OR "Randomized Controlled Trials as Topic"[Mesh] OR random*[Title/Abstract] OR "randomly assigned"[Title/Abstract] OR "controlled trial"[Title/Abstract] OR "clinical trial"[Title/Abstract] OR RCT[Title/Abstract])

Web of science:

TS=(

("child*" OR "adolescent*" OR "teenager*" OR "youth" OR "student*" OR "pediatric" OR "paediatric" OR "school-age" OR "schoolage")

AND

("myopia" OR "near-sightedness" OR "nearsightedness" OR "refractive error*" OR "axial length" OR "spherical equivalent")

AND

(“prevention” OR “management” OR “treatment” OR “therapy” OR "prevent*" OR "control*" OR "intervent*" OR "treat*" OR "management" OR "therapy" OR "correction" OR "atropine" OR "orthokeratology" OR "OK" OR "ortho-k" OR "contact lens*" OR "spectacle*" OR "glasses" OR "defocus" OR "DIMS" OR "HALT" OR "red light" OR "outdoor activity")

AND

("randomized controlled trial" OR "RCT" OR "randomized" OR "randomised" OR "placebo-controlled" OR "double-blind" OR "single-blind" OR "clinical trial")

AND

("China" OR "Chinese" OR "PRC" OR "People's Republic of China" OR "Mainland China" OR "Hong Kong" OR "Macau" OR “Taiwan”)

)

Methods S2: Construction of the State Transition Probability Matrix.

In this study, myopia is classified into three categories: mild, moderate, and severe. Each year, individuals may either maintain their current myopic state or progress to a more severe state. For instance, a patient with mild myopia at baseline may have three possible states in the second year: mild, moderate, or severe.

Let$\gamma_{1,2}$ represent the transition rate from state 1 to state 2, assuming that transition from state 2 back to state 1 is not possible. Denote P(t) as the transition probability matrix between states over a time period t, and G as the state transition rate matrix, as shown in Equation (1).

$$G = \begin{bmatrix} -(\gamma_{1,2} + \gamma_{1,3}) & \gamma_{1,2} & \gamma_{1,3} \\ 0 & -\gamma_{2,3} & \gamma_{2,3} \\ 0 & 0 & 0 \end{bmatrix} \tag{1}$$

The relationship between P(t) and G is described in Equation (2).

$$P(t) = e^{tG} = \sum_{n=0}^{\infty} \frac{t^n}{n!} G^n \tag{2}$$

The transition probability matrix among the three myopic states is shown in Equation (3).

$$\boldsymbol{P(t)} = \begin{bmatrix} \boldsymbol{e^{-\lambda_1 t}} & \dfrac{\boldsymbol{\gamma_{1,2} e^{-(\gamma_{2,3})t}\left(1 - e^{-(\lambda_1 - \gamma_{2,3})t}\right)}}{\boldsymbol{\gamma_{1,2} + \gamma_{1,3} - \gamma_{2,3}}} & \boldsymbol{\left(1 - \pi_{1,1}(t) - \pi_{1,2}(t)\right)} \\ \boldsymbol{0} & \boldsymbol{e^{-(\gamma_{2,3})t}} & \boldsymbol{\left(1 - e^{-(\gamma_{2,3})t}\right)} \\ \boldsymbol{0} & \boldsymbol{0} & \boldsymbol{1} \end{bmatrix} \tag{3}$$

Table S1. Characteristics of included studies in the network meta-analysis (SER).

Study	Treatment	Samplesize	Mean	Std.dev	Design
Bao JH 2022[1]	HAL	54	-0.33	0.05	RCT
	control	50	-0.73	0.05	RCT
Cao K 2024[2]	control	168	-0.65	0.33	RCT
	RLRL	168	0.24	0.27	RCT
Chen HY 2023[3]	RLRL	46	0.05	0.47	RCT
	control	40	-0.64	0.44	RCT
Chen XQ 2025[4]	control	72	-0.58	0.32	RCT
	CARE	74	-0.37	0.32	RCT
Chen YX 2022[5]	RLRL	31	-0.03	0.13	RCT
	0.01%Atropine	31	-0.60	0.33	RCT
Cheng D 2014[6]	control	41	-0.69	0.04	RCT
	Bifocal	48	-0.42	0.03	RCT
Cho P 2005[7]	Orthokeratology	35	1.05	0.67	RCT
	control	35	-0.60	0.31	RCT
Edwards MH 2002[8]	PAL	121	-0.56	0.34	RCT
	control	133	-0.63	0.37	RCT
Fan YZ 2025[9]	DSDO	122	-0.22	0.41	RCT
	DIMS	142	-0.29	0.46	RCT
Gao MJ 2025[10]	0.01%Atropine	83	-0.30	0.88	RCT
	PLARI	80	-0.24	0.87	RCT
	0.01%Atropine+PLARI	81	-0.15	0.64	RCT
	control	90	-0.68	1.21	RCT
He MG 2015[11]	Outdoor	919	-0.47	0.80	RCT
	control	929	-0.53	0.86	RCT
He MN 2025[12]	0.01%Atropine	32	-0.23	0.50	RCT
	control	32	-0.49	0.42	RCT
He XG 2022[13]	Outdoor	2329	-0.42	0.39	RCT
	control	2037	-0.49	0.38	RCT
He XG 2023[14]	RLRL	120	-0.35	0.54	RCT
	control	111	-0.76	0.60	RCT
Jiang Y 2022[15]	RLRL	119	-0.20	0.50	RCT
	control	145	-0.79	0.58	RCT
Jin JX 2015[16]	Outdoor	214	-0.10	0.65	RCT
	control	177	-0.27	0.52	RCT
Lam CSY 2014[17]	DISC	65	-0.30	0.25	RCT
	control	63	-0.40	0.28	RCT
Lam CSY 2020[18]	DIMS	79	-0.19	0.03	RCT
	control	81	-0.47	0.03	RCT
Lam CSY 2023[19]	DIMS	36	-0.20	0.64	RCT
	control	22	-0.50	0.66	RCT
Li XY 2025[20]	DIMS	72	-0.27	0.39	Cohort
	control	79	-0.55	0.39	Cohort
Liu GH 2024[21]	RLRL	32	-0.08	0.38	RCT
	control	36	-0.86	0.56	RCT

Liu GH 2025[22]	RLRL	130	0.18	0.63	RCT
	control	68	-0.80	0.42	RCT
Liu JX 2023[23]	DIMS	407	-0.47	0.31	RCT
	control	1118	-0.61	0.39	RCT
Liu L 2025[24]	DISC	33	-0.50	0.41	RCT
	control	35	-1.23	0.50	RCT
Liu ZZ 2024[25]	RLRL	47	-0.11	0.44	RCT
	control	47	-0.42	0.36	RCT
Lu YC 2025[26]	DSDO	121	-0.11	0.36	RCT
	0.01%Atropine+DSDO	125	-0.01	0.40	RCT
	control	124	-0.30	0.43	RCT
Peng TL 2023[27]	DISC	17	-0.69	0.69	RCT
	control	15	-1.45	0.56	RCT
Su BB 2024[28]	control	78	-0.66	0.40	RCT
	PLARI	76	-0.30	0.48	RCT
	NLARI	77	-0.21	0.35	RCT
Tan Q 2020[29]	0.01%Atropine+Orthokeratology	29	2.95	1.00	RCT
	Orthokeratology	30	2.87	0.97	RCT
Tan Q 2023[30]	0.01%Atropine+Orthokeratology	34	0.04	0.04	RCT
	Orthokeratology	35	-0.05	0.04	RCT
Wei SF 2020[31]	0.01%Atropine	76	-0.49	0.42	RCT
	control	83	-0.76	0.50	RCT
Wei SF 2023[32]	0.01%Atropine	65	-0.48	0.42	RCT
	control	68	-0.69	0.48	RCT
Wu PC 2018[33]	Outdoor	267	-0.35	0.58	RCT
	control	426	-0.47	0.74	RCT
Xiong RL 2022[34]	control	41	-0.62	0.32	RCT
	RLRL	11	-0.16	0.40	RCT
Xu Y 2024[35]	RLRL	97	0.10	0.60	RCT
	control	95	-0.75	0.65	RCT
Yam JC 2019[36]	0.05%Atropine	109	-0.27	0.61	RCT
	0.25%Atropine	108	-0.46	0.45	RCT
	0.01%Atropine	110	-0.59	0.61	RCT
	control	111	-0.81	0.53	RCT
Yam JC 2020[37]	0.05%Atropine	93	-0.28	0.43	RCT
	0.25%Atropine	86	-0.43	0.37	RCT
	0.01%Atropine	91	-0.56	0.43	RCT
Cao XD 2025[38]	0.01%Atropine+DIMS	162	-0.22	0.42	Cohort
	0.01%Atropine	75	-0.58	0.44	Cohort
Tang Y 2025[39]	HAL	62	-0.43	0.44	Cohort
	0.01%Atropine+HAL	55	-0.33	0.38	Cohort
	control	58	-0.52	0.35	Cohort
Yam JC 2023[40]	0.05%Atropine	116	-0.23	0.35	RCT
	0.01%Atropine	122	-0.42	0.40	RCT
	control	115	-0.51	0.39	RCT
Yang ZK 2009[41]	control	75	-0.75	0.34	RCT

	PAL	74	-0.62	0.28	RCT
Yi S 2015[42]	1%Atropine	68	-0.32	0.22	RCT
	control	64	-0.85	0.31	RCT
Yu J 2025[43]	RLRL	13	0.28	0.50	RCT
	PDMSL	12	-0.79	0.48	RCT
Zhou L 2023[44]	RLRL	24	0.28	0.26	RCT
	control	19	-0.97	0.25	RCT
Zhu Q 2020[45]	1%Atropine	262	-0.21	0.22	RCT
	control	308	-0.89	0.23	RCT
Zhu Q 2023[46]	0.05%Atropine	72	-0.23	0.15	RCT
	control	70	-0.86	0.56	RCT

Table S2. Characteristics of included studies in the network meta-analysis (AL).

Study	Treatment	Samplesize	Mean	Std.dev	Design
Bao JH 2022[1]	HAL	54	0.17	0.02	RCT
	control	50	0.35	0.02	RCT
Cao K 2024[2]	control	168	0.26	0.16	RCT
	RLRL	168	-0.11	0.10	RCT
Cao XD 2025[38]	0.01%Atropine+DIMS	162	0.16	0.16	Cohort
	0.01%Atropine+Orthokeratology	104	0.21	0.18	Cohort
	0.01%Atropine	75	0.40	0.18	Cohort
Charm J 2013[47]	Orthokeratology	12	0.10	0.11	RCT
	control	16	0.26	0.16	RCT
Chen CN 2013[48]	Orthokeratology	35	0.16	0.14	RCT
	control	23	0.32	0.16	RCT
Chen HY 2023[3]	RLRL	46	0.01	0.21	RCT
	control	40	0.39	0.19	RCT
Chen XQ 2025[4]	control	72	0.30	0.13	RCT
	CARE	74	0.20	0.13	RCT
Chen YX 2022[5]	RLRL	31	0.08	0.16	RCT
	0.01%Atropine	31	0.33	0.16	RCT
Cheng D 2014[6]	control	41	0.27	0.02	RCT
	Bifocal	48	0.19	0.02	RCT
Cho P 2005[7]	Orthokeratology	35	0.15	0.14	Cohort
	control	35	0.27	0.14	Cohort
Cho P 2012[49]	Orthokeratology	37	0.18	0.12	RCT
	control	41	0.32	0.13	RCT
Edwards MH 2002[8]	PAL	121	0.31	0.12	RCT
	control	133	0.32	0.14	RCT
Fan YZ 2025[9]	DSDO	122	0.16	0.17	RCT
	DIMS	142	0.20	0.22	RCT
Gao MJ 2025[10]	0.01%Atropine	83	0.14	0.56	RCT
	PLARI	80	0.10	0.68	RCT
	0.01%Atropine+PLARI	81	0.09	0.32	RCT
	control	90	0.40	0.68	RCT
He MG 2015[11]	Outdoor	919	0.32	0.23	RCT
	control	929	0.33	0.23	RCT
He MN 2025[12]	0.01%Atropine	32	0.26	0.16	RCT
	control	32	0.32	0.15	RCT
He XG 2022[13]	Outdoor	2329	0.28	0.17	RCT
	control	2037	0.31	0.17	RCT
He XG 2023[14]	RLRL	120	0.30	0.27	RCT
	control	111	0.47	0.25	RCT
Jiang Y 2022[15]	RLRL	119	0.13	0.22	RCT
	control	145	0.38	0.25	RCT
Jin JX 2015[16]	Outdoor	214	0.16	0.30	RCT
	control	177	0.21	0.21	RCT
Lam CSY 2014[17]	DISC	65	0.13	0.12	RCT

	control	63	0.19	0.12	RCT
Lam CSY 2020[18]	DIMS	79	0.11	0.01	RCT
	control	81	0.27	0.02	RCT
Lam CSY 2023[19]	DIMS	36	0.11	0.15	RCT
	control	22	0.27	0.14	RCT
Li BB 2024[50]	0.01%Atropine+Orthokeratology	26	0.10	0.13	RCT
	Orthokeratology	26	0.20	0.15	RCT
Li XY 2025[20]	DIMS	72	0.17	0.15	RCT
	control	79	0.30	0.16	RCT
Liu GH 2024[21]	RLRL	32	0.03	0.12	RCT
	control	36	0.42	0.17	RCT
Liu GH 2025[22]	RLRL	130	-0.11	0.25	RCT
	control	68	0.32	0.09	RCT
Liu L 2025[24]	DISC	33	0.22	0.13	RCT
	control	35	0.49	0.15	RCT
Liu ZZ 2024[25]	RLRL	47	0.09	0.21	RCT
	control	47	0.28	0.12	RCT
Lu YC 2025[26]	DSDO	121	0.16	0.11	RCT
	0.01%Atropine+DSDO	125	0.14	0.14	RCT
	control	124	0.27	0.14	RCT
Peng TL 2023[27]	DISC	17	0.25	0.22	RCT
	control	15	0.43	0.16	RCT
Song DS 2025[51]	0.01%Atropine+DIMS	88	0.14	0.21	RCT
	Orthokeratology	73	0.20	0.16	RCT
Su BB 2024[28]	control	78	0.34	0.18	RCT
	PLARI	76	0.19	0.20	RCT
	NLARI	77	0.17	0.14	RCT
Tan Q 2020[29]	0.01%Atropine+Orthokeratology	29	0.07	0.16	RCT
	Orthokeratology	30	0.16	0.15	RCT
Tan Q 2023[30]	0.01%Atropine+Orthokeratology	34	0.09	0.02	RCT
	Orthokeratology	35	0.17	0.02	RCT
Tang T 2024[52]	DIMS	41	0.22	0.14	Cohort
	0.01%Atropine+DIMS	42	0.15	0.15	Cohort
	Orthokeratology	41	0.20	0.12	Cohort
	0.01%Atropine+Orthokeratology	43	0.12	0.14	Cohort
Tang Y 2025[39]	HAL	62	0.21	0.15	Cohort
	0.01%Atropine+HAL	55	0.21	0.13	Cohort
	control	58	0.28	0.15	Cohort
Wang H 2025[53]	Orthokeratology	45	0.21	0.09	Cohort
	0.01%Atropine+Orthokeratology	45	0.12	0.08	Cohort
Wei SF 2020[31]	0.01%Atropine	76	0.32	0.19	RCT
	control	83	0.41	0.19	RCT
Wei SF 2023[32]	0.01%Atropine	65	0.30	0.20	RCT
	control	68	0.37	0.18	RCT
Wu PC 2018[33]	Outdoor	267	0.28	0.22	RCT
	control	426	0.33	0.35	RCT

Xiong RL 2022[34]	control	41	0.32	0.15	RCT
	RLRL	11	0.08	0.19	RCT
Xu SS 2023[54]	control	30	0.27	0.12	RCT
	0.01%Atropine	31	0.14	0.08	RCT
	Orthokeratology	34	0.14	0.07	RCT
	0.01%Atropine+Orthokeratology	34	0.10	0.08	RCT
Xu Y 2024[35]	RLRL	97	-0.06	0.20	RCT
	control	95	0.34	0.22	RCT
Yam JC 2019[36]	0.05%Atropine	109	0.20	0.25	RCT
	0.25%Atropine	108	0.29	0.20	RCT
	0.01%Atropine	110	0.36	0.29	RCT
	control	111	0.41	0.22	RCT
Yam JC 2020[37]	0.05%Atropine	93	0.20	0.18	RCT
	0.25%Atropine	86	0.25	0.17	RCT
	0.01%Atropine	91	0.30	0.19	RCT
	control	80	0.43	0.21	RCT
Yam JC 2023[40]	0.05%Atropine	116	0.24	0.15	RCT
	0.01%Atropine	122	0.32	0.19	RCT
	control	115	0.35	0.17	RCT
Yi S 2015[42]	1%Atropine	68	-0.03	0.07	RCT
	control	64	0.32	0.15	RCT
Yu J 2025[43]	RLRL	13	-0.18	0.17	RCT
	PDMSL	12	0.34	0.13	RCT
Yu SA 2022[55]	0.01%Atropine+Orthokeratology	27	0.10	0.14	RCT
	Orthokeratology	26	0.20	0.15	RCT
Zhao YX 2024[56]	0.01%Atropine+HAL	35	0.09	0.11	Cohort
	HAL	35	0.19	0.16	Cohort
	control	35	0.34	0.16	Cohort
Zhou L 2023[44]	RLRL	24	-0.02	0.11	RCT
	control	19	0.48	0.16	RCT
Zhu MJ 2022[57]	Orthokeratology	95	0.25	0.27	RCT
	control	88	0.44	0.23	RCT
Zhu Q 2020[45]	1%Atropine+PAL	262	0.12	0.10	RCT
	PAL	308	0.40	0.11	RCT
Zhu Q 2023[46]	0.05%Atropine	72	0.13	0.15	RCT
	control	70	0.38	0.31	RCT

Table S3. Sources of literature on transition probabilities.

Study	Location	Follow up months	Treat	Age	Boy(%)	N	Baseline SER	Annual change in SER	Baseline AL	Annual change in AL
Liu JX 2023[23]	Multicenter	24	DIMS	6-16	52.9	2629	-2.93±1.69	-0.5±0.43	NA	NA
		24	SVL	6-16	51.1	6838	-2.7±1.76	-0.77±0.58	NA	NA
Yam JC 2019[36]	Hong Kong	12	0.05% Atropine	8.45 ± 1.81	49.5	109	-3.98±1.69	-0.27±0.61	24.85±0.9	0.20±0.25
		12	0.25% Atropine	8.54 ± 1.71	60.2	108	-3.71±1.85	-0.46±0.45	24.86±0.95	0.29±0.2
		12	0.01% Atropine	8.23 ± 1.83	57.3	110	-3.77±1.85	-0.59±0.61	24.7±0.99	0.36±0.29
		12	placebo	8.42 ± 1.72	59.5	111	-3.85±1.95	-0.81±0.53	24.82±0.97	0.41±0.22
Wei SF 2020[31]	Beijing	12	0.01% Atropine	9.44 ± 1.8	56	76	-2.52±1.33	-0.49±0.42	24.50±0.76	0.32±0.19
		12	placebo	9.84 ± 1.53	61	83	-2.64±1.46	-0.76±0.5	24.69±0.97	0.41±0.19
Bao JH 2022[1]	Wenzhou	24	HAL	10.6±0.2	48	54	-2.7±0.14	-0.33±0.045	24.76±0.09	0.17±0.02
		24	SAL	10.2±0.2	32	53	-2.28±0.13	-0.52±0.03	24.44±0.1	0.26±0.02
		24	SVL	10.4±0.2	58	50	-2.44±0.12	-0.73±0.045	24.77±0.09	0.35±0.02
Tang Y 2025[39]	Wenzhou	12	HAL	10.37 ± 1.36	56.45	62	-5.2±0.99	-0.43±0.44	25.6±0.74	0.21±0.15
		12	0.01% Atropine + HAL	10.16 ± 1.24	49.09	55	-5.22±0.81	-0.33±0.38	25.42±0.86	0.21±0.13
		12	SVL	10.28 ± 1.20	53.45	58	-4.96±0.9	-0.52±0.35	25.39±0.78	0.28±0.15
Lam CSY 2020[18]	Hong Kong	24	DIMS	10.20±1.47	58.2	79	-2.97±0.97	-0.19±0.03	24.7±0.82	0.11±0.01
		24	SVL	10.00±1.45	54.3	81	-2.76±0.96	-0.47±0.03	24.6±0.83	0.27±0.02
Li XY 2025[20]	Chengdu	12	DIMS	10.69 (10.33~11.06)	59.7	72	-3.16 (-3.54~-2.78)	-0.27±0.39	24.82 (24.62~25.03)±	0.17±0.15

		12	SVL	10.38 (10.00~10.76)	55.7	79	-3.13 (-3.49~-2.77)	-0.55±0.39	25.02 (24.79~25.26)	0.30±0.16
He MG 2015[11]	Guangzhou	36	outdoor	6.61±0.33	52.58	919	1.3±0.97	-0.47±0.8	22.6±0.71	0.32±0.23
		36	control	6.57±0.32	54.61	929	1.26±0.81	-0.53±0.86	22.66±0.7	0.33±0.23
Lam CSY 2014[17]	Hong Kong	24	DISC	11.06 ± 1.55	32.3	65	-2.9±1.05	-0.30±0.25	24.69±0.74	0.13±0.12
		24	SVL	10.87 ± 1.67	38.1	63	-2.8±1.03	-0.40±0.28	24.62±0.79	0.19±0.12
Liu GH 2024[21]	Tianjin	12	RLRL	9.37±1.69	50	32	-2.91±1.27	-0.08±0.38	24.71±0.92	0.03±0.12
		12	control	9.55±1.13	47.22	36	-2.61±0.98	-0.86±0.56	24.58±0.64	0.42±0.17
Chen YX 2022[5]	Shenzhen	12	RLRL	9.78±1.58	45.2	31	-2.6±1.17	-0.03±0.13	24.48±0.79	0.08±0.16
		12	0.01% Atropine	10.31±1.90	57.5	31	-2.59±1.24	-0.6±0.33	24.67±0.98	0.33±0.16
Yu SA 2022[55]	Zhengzhou	12	0.01% Atropine + orthokeratology	10.07 ± 1.44	37.04	27	-2.81±0.92	NA	24.79±0.72	0.10±0.14
		12	orthokeratology	9.80 ± 1.64	57.69	26	-2.81±0.97	NA	24.64±0.79	0.20±0.15
Chen XQ 2025[4]	multicenter:Tianjin, Shenyang, Beijing	24	SVL	9.8 ± 1.5	50	72	-2.31±0.75	-0.58±0.32	24.45±0.75	0.30±0.13
		24	CARE 7mm	9.7 ± 1.7	50	74	-2.31±1.07	-0.37±0.32	24.31±0.71	0.20±0.13
		24	CARE S 9mm	10.0 ± 1.7	56.9	72	-2.24±0.96	-0.40±0.28	24.44±0.69	0.22±0.13
Edwards MH 2002[8]	Hong Kong	36	PAL	8.92(7 to 10.5)	50.4	121	-2.82±0.9	-0.56±0.34	24.4±0.69	0.31±0.12
		36	SVL	9.17(7 to 10.5)	45.9	133	-2.92±0.99	-0.63±0.37	24.44±0.77	0.32±0.14
Yang ZK 2009	Guangzhou	24	SVL	NA	58.7	75	-1.78±0.68	-1.50±0.67	NA	NA
		24	PAL	NA	45.9	74	-1.6±0.63	-1.24±0.56	NA	NA
Zhu Q 2020[45]	Shanghai	12	RLRL	10.54 ± 2.03	69.23	13	-5.36±1.32	0.28±0.5	25.74±0.88	-0.18±0.17

		12	PDMSL	10.41 ± 1.78	58.33	12	-4.73±1.08	-0.79±0.48	25.59±0.91	0.34±0.13
Yu J 2025[43]	Wenzhou	12	DSDO	7.05±0.12	51.3	121	0.5±0.39	-0.11±0.36	23.18±0.06	0.16±0.11
		12	0.01% Atropine + DSDO	7.02±0.14	48.7	125	0.51±0.41	-0.01±0.4	23.09±0.06	0.14±0.14
Lu YC 2025[26]		12	SVL	7.01±0.11	42	124	0.54±0.36	-0.3±0.43	23.13±0.06	0.27±0.14
	Beijing	12	DSDO	9.35±2.01	53.28	122	-1.84±1.36	-0.22±0.41	24.19±1.01	0.16±0.17
		12	DIMS	9.24±1.77	53.52	142	-1.78±1.1	-0.29±0.46	24.21±0.87	0.2±0.22
Luo YH 2025[58]	Shenyang	12	0.01% Atropine + DIMS	8.48±1.45	51.2	162	-1.63±0.71	-0.22±0.42	24.08±0.71	0.16±0.16
		12	0.01% Atropine + orthokeratology	9.64±1.02	59	104	-2.16±0.7	NA	24.42±0.91	0.21±0.18
Cao XD 2025[38]		12	0.01% Atropine + SVL	8.63±1.00	40	75	-1.14±0.84	-0.58±0.44	23.79±0.54	0.40±0.18
	Wenzhou	12	HAL	10.37 ± 1.36	56.45	62	-5.2±0.99	-0.43±0.44	25.6±0.74	0.21±0.15
		12	0.01% Atropine + HAL	10.16 ± 1.24	49.09	55	-5.22±0.81	-0.33±0.38	25.42±0.86	0.21±0.13
Tang Y 2025[39]		12	SVL	10.28 ± 1.20	53.45	58	-4.96±0.9	-0.52±0.35	25.39±0.78	0.28±0.15
	Hong Kong	24	0.01% Atropine + orthokeratology	9.2 ± 1.0	44.1	34	-2.76±0.88	0.04±0.04	24.56±0.71	0.09±0.02
		24	orthokeratology	9.1 ± 1.2	42.9	35	-2.83±1.01	-0.05±0.04	24.5±0.92	0.17±0.02
Tan Q 2023[30]	Multicenter	24	DIMS	6-16	52.9	2629	-2.93±1.69	-0.5±0.43	NA	NA
		24	SVL	6-16	51.1	6838	-2.7±1.76	-0.77±0.58	NA	NA

Table S4. CINeMA Confidence Assessment for Network Meta-Analysis Estimates.

Comparison (vs SVL)	RCT	Cohort	Total	Within-study bias	Reporting bias	Indirectness	Imprecision	Heterogeneity	Incoherence	Overall confidence
**SER (D)**										
RLRL vs SVL	12	0	12	No concerns	No concerns	No concerns	No concerns	Some concerns	No concerns	Moderate
0.01%Atr vs SVL	9	0	9	No concerns	No concerns	No concerns	No concerns	Some concerns	No concerns	Moderate
0.01%Atr + DIMS vs SVL	0	1	1	Some concerns	Some concerns	Some concerns	No concerns	No concerns	No concerns	Moderate
DIMS vs SVL	4	1	5	No concerns	Some concerns	No concerns	No concerns	Some concerns	No concerns	Moderate
HAL vs SVL	1	1	2	No concerns	Some concerns	No concerns	Some concerns	No concerns	No concerns	Moderate
DSDO vs SVL	2	0	2	No concerns	Some concerns	No concerns	Some concerns	No concerns	No concerns	Moderate
DISC vs SVL	3	0	3	No concerns	Some concerns	No concerns	No concerns	No concerns	No concerns	Moderate
Outdoor vs SVL	4	0	4	No concerns	Some concerns	No concerns	Some concerns	Some concerns	No concerns	Moderate
0.01%Atr + DSDO vs SVL	1	0	1	No concerns	Some concerns	No concerns	Some concerns	No concerns	No concerns	Moderate
0.01%Atr + HAL vs SVL	0	1	1	Some concerns	Some concerns	No concerns	Some concerns	No concerns	No concerns	Moderate
0.01%Atr + Orthokeratology vs SVL	2	0	2	Some concerns	Some concerns	Some concerns	No concerns	No concerns	No concerns	Moderate
Orthokeratology vs SVL	2	0	2	Some concerns	Some concerns	No concerns	No concerns	No concerns	No concerns	Moderate
**AL (mm)**										
RLRL vs SVL	12	0	12	No concerns	No concerns	No concerns	No concerns	Some concerns	No concerns	Moderate
0.01%Atr vs SVL	9	1	10	No concerns	No concerns	No concerns	No concerns	Some concerns	No concerns	Moderate
0.01%Atr + DIMS vs SVL	0	3	3	Some concerns	Some concerns	Some concerns	No concerns	No concerns	No concerns	Moderate
DIMS vs SVL	4	1	5	No concerns	Some concerns	No concerns	No concerns	No concerns	No concerns	Moderate
HAL vs SVL	1	2	3	No concerns	Some concerns	No concerns	No concerns	No concerns	No concerns	Moderate
DSDO vs SVL	2	0	2	No concerns	Some concerns	No concerns	No concerns	No concerns	No concerns	Moderate
DISC vs SVL	3	0	3	No concerns	Some concerns	No concerns	No concerns	No concerns	No concerns	Moderate
Outdoor vs SVL	4	0	4	No concerns	Some concerns	No concerns	Some concerns	Some concerns	No concerns	Moderate
Orthokeratology vs SVL	5	1	6	Some concerns	No concerns	No concerns	No concerns	Some concerns	No concerns	Moderate
0.01%Atr + Orthokeratology vs SVL	8	0	8	No concerns	Some concerns	No concerns	No concerns	No concerns	No concerns	Moderate

Note: Confidence was assessed across six domains using the CINeMA (Confidence in Network Meta-Analysis) framework. For each domain, concerns were rated as "no concerns", "some concerns", or "major concerns". The overall confidence in each comparison was rated as high, moderate, low, or very low. Within-study bias was assessed using the Cochrane Risk of Bias tool for 51 RCTs and the Newcastle-Ottawa Scale (NOS) for 7 cohort studies. "RCT" = number of RCTs with direct comparison vs SVL; "Cohort" = number of cohort studies; "Total" = total studies

in the network involving that intervention. Within-study bias was assessed using the Cochrane Risk of Bias tool (7 domains) for 51 RCTs and the Newcastle-Ottawa Scale (NOS, 8 domains) for 7 cohort studies. All 7 cohort studies exhibited high risk for representativeness (hospital-based volunteer cohorts rather than community-based randomly selected individuals); however, all other NOS domains (ascertainment of exposure, assessment of outcome, comparability, follow-up adequacy) were rated as low risk. For comparisons involving cohort studies, the within-study bias rating reflects the combined assessment of both RCT and cohort evidence. Comparisons that relied entirely on cohort studies (e.g., 0.01% atropine + DIMS for SER) were rated as "some concerns" for within-study bias. The overall confidence was rated as moderate for all key comparisons. No comparisons were rated as low or very low confidence.

Table S5. Mixed NMA vs RCT-only NMA: Comparison of Effect Estimates (vs SVL control).

Intervention	Mixed NMA (TE)	RCT-only NMA (TE, 95% CI)	Difference (%)	Evidence Source
**SER (D)**				
RLRL	0.74 (0.62, 0.85)	0.74 (0.61, 0.86)	0%	RCT + Cohort
DIMS	0.23 (0.04,0.41)	0.21 (-0.004, 0.42)	-8.70%	RCT + Cohort
HAL	0.26 (-0.01, 0.52)	0.40 (0.04, 0.76)	53.80%	RCT + Cohort
0.01%AT	0.22 (0.08, 0.37)	0.23 (0.08, 0.37)	4.50%	RCT + Cohort
DISC	0.45 (0.20, 0.70)	0.44 (0.20, 0.71)	-2.20%	RCT + Cohort
Outdoor	0.10 (-0.08, 0.29)	0.10 (-0.08, 0.29)	0%	RCT + Cohort
OK	1.60 (1.20, 2.10)	1.60 (1.20, 2.10)	0%	RCT + Cohort
DSDO	0.24 (-0.04, 0.52)	0.23 (-0.06, 0.52)	-4.20%	RCT + Cohort
0.01%AT+DSDO	0.32 (-0.04, 0.67)	0.31 (-0.04, 0.67)	-3.10%	RCT + Cohort
0.01%AT+DIMS	0.59 (0.18, 0.99)	Not estimable	-	Cohort only
0.01%AT+HAL	0.27 (-0.09, 0.62)	Not estimable	-	Cohort only
**AL (mm)**				
RLRL	-0.33 (-0.38, -0.29)	-0.33 (-0.38, -0.29)	0%	RCT + Cohort
DIMS	-0.14 (-0.20, -0.08)	-0.14 (-0.21, -0.04)	0%	RCT + Cohort
HAL	-0.13 (-0.21, -0.06)	-0.18 (-0.29, -0.05)	38.50%	RCT + Cohort
0.01%AT	-0.09 (-0.13, -0.05)	-0.10 (-0.14, -0.05)	10.20%	RCT + Cohort
DISC	-0.16 (-0.24, -0.08)	-0.16 (-0.24, -0.08)	0%	RCT + Cohort
Outdoor	-0.03 (-0.10, 0.03)	-0.03 (-0.10, 0.03)	0%	RCT + Cohort
OK	-0.15 (-0.19, -0.10)	-0.14 (-0.19, -0.08)	-6.70%	RCT + Cohort
DSDO	-0.14 (-0.23, -0.05)	-0.14 (-0.24, -0.05)	0%	RCT + Cohort
0.01%AT+DSDO	-0.15 (-0.26, -0.03)	-0.15 (-0.27, -0.03)	0%	RCT + Cohort
0.01%AT+DIMS	-0.24 (-0.31, -0.16)	-0.20 (-0.34, -0.05)	-16.70%	RCT + Cohort
0.01%AT+HAL	-0.17 (-0.26, -0.08)	Not estimable	-	Cohort only

Note: The mixed NMA included both RCTs and cohort studies. The RCT-only NMA was restricted to studies with design = RCT. Treatment effect (TE) estimates are mean differences (positive = intervention better than SVL for SER; negative = intervention better than SVL for AL). "Not estimable" indicates that the intervention was not present in the RCT-only network (i.e., evidence came exclusively from cohort studies). For SER, 0.01% atropine + DIMS and 0.01% atropine + HAL relied entirely on cohort studies.

For AL, 0.01% atropine + HAL relied entirely on cohort studies. For all other interventions, RCT-only estimates were consistent with mixed NMA estimates (difference <10%), confirming the robustness of the core findings.

Table S6. Simulation results for the proportion of high myopia in an 8-year-old population with newly diagnosed myopia.

Myopia Prevention and Control Measures	Proportion of High Myopia After 8 Years	Improvement over SVL	Improvement over monotherapy	Improvement over 0.01%Atr
SVL	75.00%			
0.01%AT+DSDO	1.52%	73.48%	27.32%	59.04%
0.01%AT+OK	26.16%	48.84%	8.57%	34.40%
0.01%AT+DIMS	27.00%	48.00%	0.58%	33.56%
DSDO	28.84%	46.16%		
DIMS	27.59%	47.41%		
OK	34.74%	40.27%		
0.01%AT+HAL	41.34%	33.66%	3.97%	19.22%
HAL	45.31%	29.69%		
0.01%AT	60.57%	14.43%		

Table S7. Average annual costs ($) of myopia control interventions by regional economic development level.

Myopia control interventions	The national average	High-income regions	Lower-income regions
SVL	156.99	196.83	120.15
Outdoor	156.99	196.83	120.15
DIMS	493.36	513.83	472.38
HAL	496.01	536.93	458.44
DSDO	571.46	581.33	549.25
CARE	483.77	479.12	476.73
PDMSL	315.57	306.69	310.41
PAL	350.03	380.43	317.73
0.01% Atr	619.52	719.02	521.84
DISC	1566.61	1461.17	1672.05
OK	1090.49	1404.23	894.20
RLRL	1544.81	1836.36	1268.60
0.01% Atr + DIMS	1082.03	1183.16	947.89
0.01% Atr + HAL	1084.69	1206.26	933.94
0.01% Atr + DSDO	1160.14	1250.66	1024.75
0.01% Atr + OK	1673.42	2033.09	1325.24

Table S8. Cost-effectiveness results of myopia control interventions for cohorts with onset at age 6.

Strategy	Cost	Incr Cost	Eff	Incr Eff	ICER
SER					
SVL	1234.69		-5.80		
**Outdoor**	**1110.72**	**-123.97**	**0.74**	**6.54**	**-18.96**
PDMSL	2375.41	1140.72	-2.32	3.48	328.22
PAL	2583.42	1348.73	0.68	6.48	208.29
CARE	3536.63	2301.94	1.46	7.26	317.01
DIMS	3650.19	2415.51	1.64	7.44	324.85
HAL	3659.34	2424.65	1.84	7.64	317.52
DSDO	4191.43	2956.74	1.67	7.47	395.84
0.01%Atr	4564.80	3330.11	1.54	7.33	454.08
**0.01%Atr+DIMS**	**7772.22**	**6537.53**	**4.33**	**10.13**	**645.68**
0.01%Atr+HAL	7821.41	6586.72	1.87	7.67	858.58
0.01%Atr+DSDO	8346.01	7111.32	2.29	8.09	879.18
**RLRL**	**11095.22**	**9860.53**	**5.26**	**11.06**	**891.34**
DISC	11387.31	10152.62	3.20	9.00	1127.96
AL					
SVL	1230.69		-2.57		
**Outdoor**	**1109.78**	**-120.90**	**0.20**	**2.77**	**-43.68**
PDMSL	2380.80	1150.12	-1.34	1.23	937.00
PAL	2595.00	1364.32	0.09	2.65	514.35
CARE	3568.10	2337.41	0.72	3.28	712.33
**DIMS**	**3622.63**	**2391.94**	**1.00**	**3.57**	**670.64**
HAL	3640.29	2409.61	0.92	3.49	690.94
DSDO	4157.15	2926.47	1.00	3.57	820.25
0.01%Atr	4532.68	3301.99	0.64	3.21	1029.82
0.01%Atr+DIMS	7771.14	6540.45	1.71	4.28	1529.00
0.01%Atr+HAL	7774.08	6543.39	1.19	3.76	1740.41
OK	7849.73	6619.04	1.07	3.64	1820.81
0.01%Atr+DSDO	8341.85	7111.17	1.10	3.66	1940.86
**RLRL**	**11188.84**	**9958.15**	**2.35**	**4.92**	**2025.57**
DISC	11405.34	10174.66	1.13	3.69	2755.07
0.01%Atr+OK	12106.99	10876.30	1.57	4.13	2632.32

Table S9. Cost-effectiveness results of myopia control interventions for cohorts with onset at age 10.

Strategy	Cost	Incr Cost	Eff	Incr Eff	ICER
SER					
SVL	1235.35		-5.78		
**Outdoor**	**1118.85**	**-116.50**	**0.75**	**6.54**	**-17.82**
PDMSL	2377.17	1141.82	-2.49	3.29	346.95
PAL	2617.75	1382.40	0.70	6.48	213.35
CARE	3581.84	2346.49	1.51	7.30	321.47
HAL	3622.88	2387.53	1.90	7.69	310.51
DIMS	3638.32	2402.97	1.65	7.43	323.36
DSDO	4198.77	2963.42	1.71	7.49	395.56
0.01%Atr	4501.72	3266.37	1.61	7.39	441.85
**0.01%Atr+DIMS**	**7815.27**	**6579.92**	**4.26**	**10.04**	**655.28**
0.01%Atr+HAL	7819.53	6584.18	1.94	7.72	852.73
0.01%Atr+DSDO	8393.27	7157.92	2.25	8.04	890.64
**RLRL**	**11151.17**	**9915.82**	**5.28**	**11.06**	**896.35**
DISC	11199.93	9964.59	3.19	8.98	1109.91
AL					
SVL	1240.83		-2.57		
**Outdoor**	**1126.17**	**-114.66**	**0.21**	**2.78**	**-41.23**
PDMSL	2366.07	1125.25	-1.35	1.22	921.78
PAL	2610.82	1369.99	0.07	2.64	518.75
CARE	3588.71	2347.88	0.68	3.25	722.92
**DIMS**	**3619.69**	**2378.86**	**1.00**	**3.57**	**666.34**
HAL	3685.47	2444.64	0.93	3.50	698.09
DSDO	4226.83	2986.00	1.02	3.59	832.43
0.01%Atr	4529.42	3288.60	0.65	3.22	1020.83
0.01%Atr+HAL	7810.83	6570.00	1.22	3.79	1731.82
0.01%Atr+DIMS	7874.96	6634.14	1.72	4.29	1546.98
OK	7913.03	6672.21	1.08	3.65	1828.33
0.01%Atr+DSDO	8269.06	7028.23	1.06	3.64	1933.40
**RLRL**	**11210.79**	**9969.96**	**2.35**	**4.92**	**2025.17**
DISC	11398.45	10157.62	1.15	3.72	2728.57
0.01%Atr+OK	12063.88	10823.06	1.58	4.15	2608.72

Table S10. Cost-effectiveness results of myopia control interventions for cohorts with onset at age 12.

Strategy	Cost	Incr Cost	Eff	Incr Eff	ICER
SER					
SVL	1235.60		-5.77		
**Outdoor**	**1113.16**	**-122.44**	**0.75**	**6.53**	**-18.76**
PDMSL	2360.31	1124.71	-2.38	3.39	331.87
PAL	2637.39	1401.79	0.73	6.50	215.64
CARE	3595.09	2359.49	1.51	7.28	324.20
DIMS	3608.88	2373.28	1.59	7.37	322.20
HAL	3642.00	2406.40	1.87	7.64	314.78
DSDO	4174.63	2939.03	1.76	7.53	390.06
0.01%Atr	4520.06	3284.46	1.57	7.35	447.14
**0.01%Atr+DIMS**	**7819.57**	**6583.97**	**4.14**	**9.92**	**663.99**
0.01%Atr+HAL	7880.45	6644.85	1.94	7.72	861.28
0.01%Atr+DSDO	8401.46	7165.86	2.28	8.05	889.68
**RLRL**	**11111.23**	**9875.62**	**5.28**	**11.06**	**893.19**
DISC	11268.41	10032.81	3.16	8.93	1123.23
AL					
SVL	1249.39		-2.56		
**Outdoor**	**1130.05**	**-119.34**	**0.22**	**2.79**	**-42.80**
PDMSL	2377.69	1128.30	-1.35	1.21	930.53
PAL	2616.58	1367.19	0.08	2.64	517.25
CARE	3577.92	2328.53	0.74	3.30	705.43
**DIMS**	**3599.24**	**2349.85**	**0.98**	**3.55**	**662.31**
HAL	3682.71	2433.31	0.93	3.49	697.21
DSDO	4188.13	2938.74	0.97	3.53	831.64
0.01%Atr	4562.92	3313.52	0.63	3.20	1035.76
0.01%Atr+HAL	7812.06	6562.67	1.21	3.77	1739.51
OK	7840.40	6591.01	1.07	3.63	1815.50
0.01%Atr+DIMS	7919.51	6670.12	1.72	4.28	1558.04
0.01%Atr+DSDO	8411.05	7161.66	1.10	3.66	1954.18
**RLRL**	**11224.46**	**9975.07**	**2.35**	**4.92**	**2027.64**
DISC	11256.07	10006.67	1.14	3.71	2699.22
0.01%Atr+OK	12070.05	10820.65	1.56	4.12	2624.48

Table S11. Cost-effectiveness results of myopia control interventions in less economically developed regions.

Strategy	Cost	Incr Cost	Eff	Incr Eff	ICER
SER					
SVL	974.6228		-5.77124		
**Outdoor**	**856.032**	**-118.59**	**0.712499**	**6.48**	**-18.29**
PDMSL	2330.229	1355.61	-2.4225	3.35	404.81
PAL	2382.399	1407.78	0.712499	6.48	217.12
HAL	3384.955	2410.33	1.852497	7.62	316.16
DIMS	3484.328	2509.70	1.638747	7.41	338.69
CARE	3515.242	2540.62	1.496247	7.27	349.59
0.01%Atr	3836.701	2862.08	1.567497	7.34	390.00
DSDO	4031.978	3057.36	1.709997	7.48	408.67
0.01%Atr+HAL	6772.932	5798.31	1.923747	7.69	753.52
**0.01%Atr+DIMS**	**6872.304**	**5897.68**	**4.203743**	**9.97**	**591.25**
0.01%Atr+DSDO	7419.954	6445.33	2.279996	8.05	800.54
**RLRL**	**9157.318**	**8182.70**	**5.272491**	**11.04**	**740.94**
DISC	12031.91	11057.29	3.206244	8.98	1231.67
AL					
SVL	974.62		-2.56		
**Outdoor**	**856.03**	**-118.59**	**0.21**	**2.78**	**-42.68**
PDMSL	2330.23	1355.61	-1.35	1.21	1119.18
PAL	2382.40	1407.78	0.07	2.64	534.01
HAL	3384.96	2410.33	0.93	3.49	690.39
**DIMS**	**3484.33**	**2509.70**	**1.00**	**3.56**	**704.48**
CARE	3515.24	2540.62	0.71	3.28	775.17
0.01%Atr	3836.70	2862.08	0.64	3.21	892.66
DSDO	4031.98	3057.36	1.00	3.56	858.21
OK	6489.78	5515.16	1.07	3.63	1517.76
0.01%Atr+HAL	6772.93	5798.31	1.21	3.78	1535.47
0.01%Atr+DIMS	6872.30	5897.68	1.71	4.27	1379.58
0.01%Atr+DSDO	7419.95	6445.33	1.07	3.63	1773.74
**RLRL**	**9157.32**	**8182.70**	**2.35**	**4.92**	**1664.42**
0.01%Atr+OK	9560.94	8586.32	1.57	4.13	2077.76
DISC	12031.91	11057.29	1.14	3.70	2984.43

Table S12. Cost-effectiveness results of myopia control interventions in highly developed regions.

Strategy	Cost	Incr Cost	Eff	Incr Eff	ICER
SER					
SVL	1521.01		-5.77		
**Outdoor**	**1402.42**	**-118.59**	**0.71**	**6.48**	**-18.29**
PDMSL	2303.76	782.74	-2.42	3.35	233.74
PAL	2829.18	1308.17	0.71	6.48	201.76
CARE	3532.30	2011.29	1.50	7.27	276.75
DIMS	3779.57	2258.56	1.64	7.41	304.80
HAL	3944.20	2423.19	1.85	7.62	317.85
DSDO	4260.53	2739.52	1.71	7.48	366.19
0.01%Atr	5241.57	3720.56	1.57	7.34	506.97
**0.01%Atr+DIMS**	**8548.56**	**7027.55**	**4.20**	**9.97**	**704.52**
0.01%Atr+HAL	8713.19	7192.18	1.92	7.69	934.66
0.01%Atr+DSDO	9029.53	7508.51	2.28	8.05	932.59
DISC	10529.40	9008.39	3.21	8.98	1003.44
**RLRL**	**13202.60**	**11681.59**	**5.27**	**11.04**	**1057.76**
AL					
SVL	1521.01		-2.56		
**Outdoor**	**1402.42**	**-118.59**	**0.21**	**2.78**	**-42.68**
PDMSL	2303.76	782.74	-1.35	1.21	646.23
PAL	2829.18	1308.17	0.07	2.64	496.22
CARE	3532.30	2011.29	0.71	3.28	613.67
**DIMS**	**3779.57**	**2258.56**	**1.00**	**3.56**	**633.98**
HAL	3944.20	2423.19	0.93	3.49	694.08
DSDO	4260.53	2739.52	1.00	3.56	768.99
0.01%Atr	5241.57	3720.56	0.64	3.21	1160.41
**0.01%Atr+DIMS**	**8548.56**	**7027.55**	**1.71**	**4.27**	**1643.87**
0.01%Atr+HAL	8713.19	7192.18	1.21	3.78	1904.59
0.01%Atr+DSDO	9029.53	7508.51	1.07	3.63	2066.33
OK	10123.71	8602.70	1.07	3.63	2367.45
DISC	10529.40	9008.39	1.14	3.70	2431.42
**RLRL**	**13202.60**	**11681.59**	**2.35**	**4.92**	**2376.12**
0.01%Atr+OK	14604.31	13083.30	1.57	4.13	3165.96

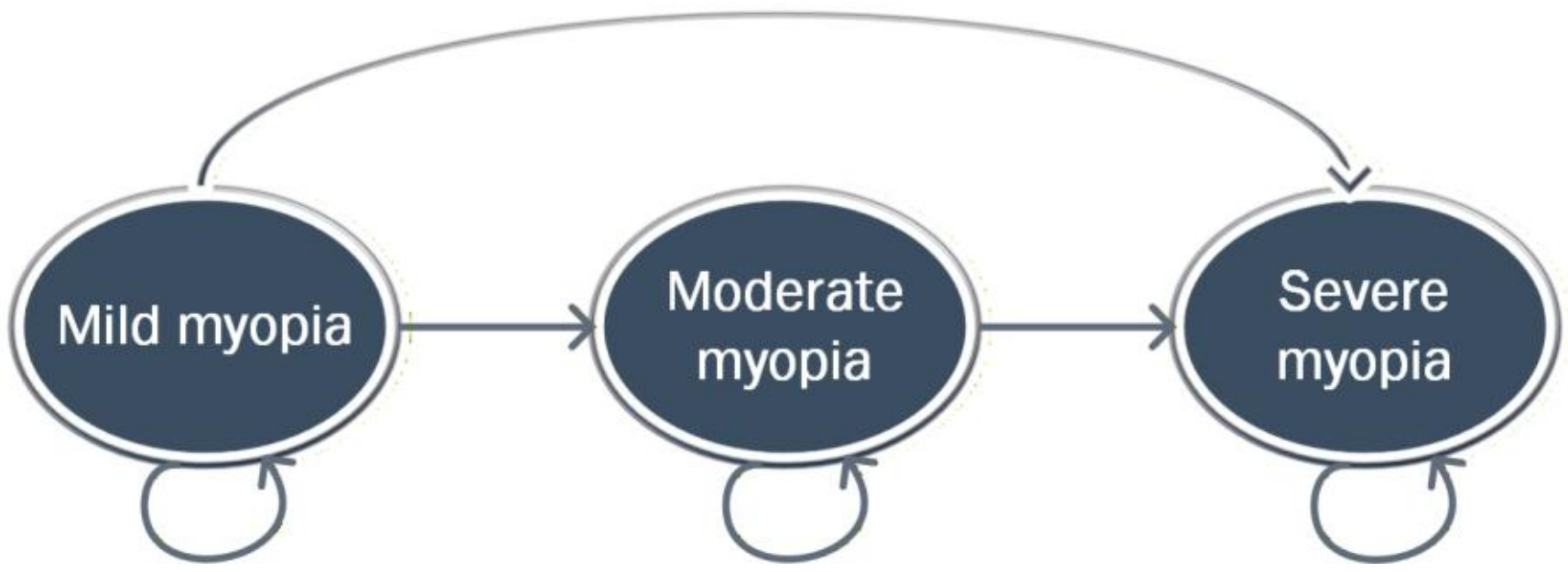

Figure S1. Markov chain of myopia state transitions.

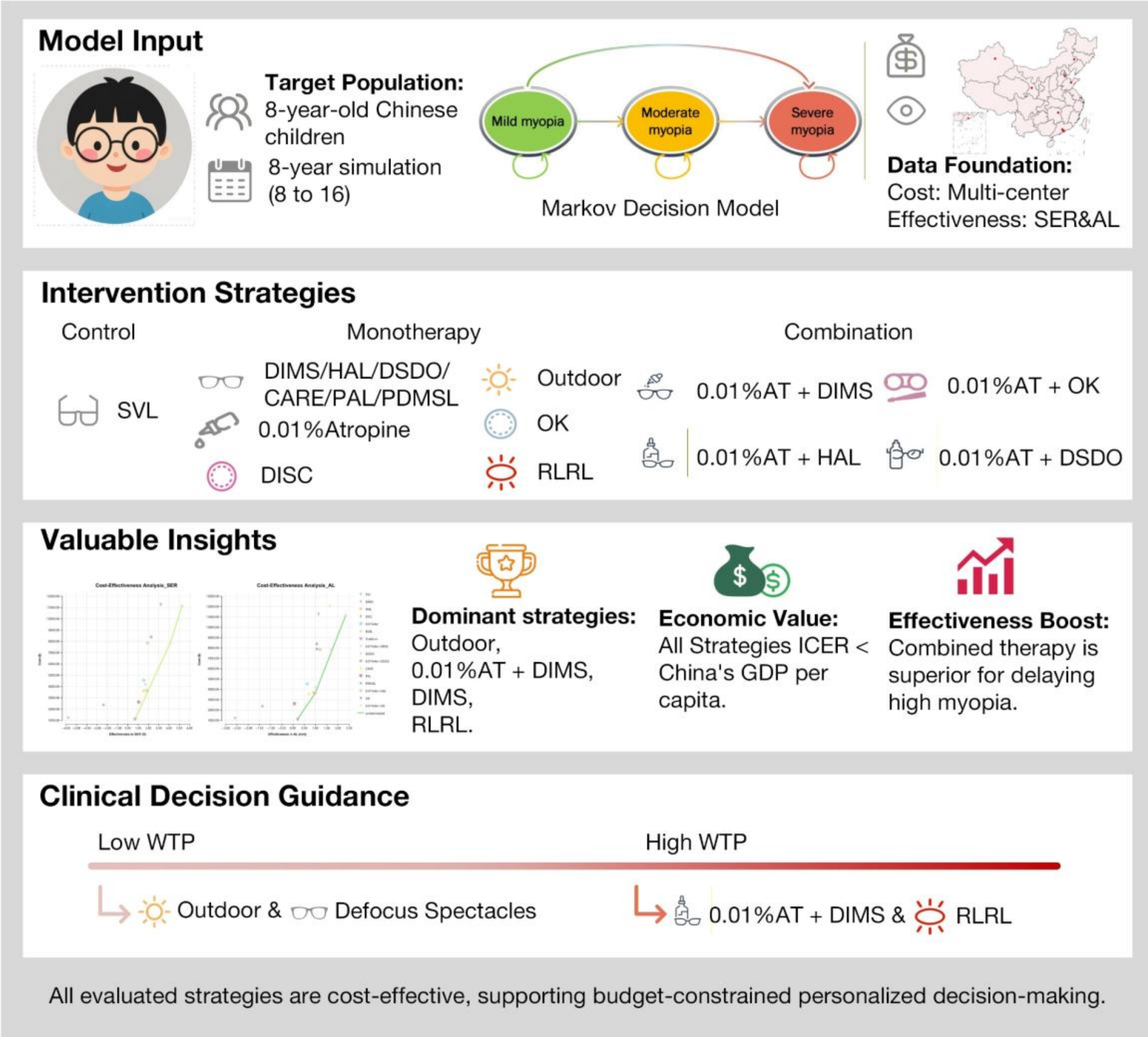

Figure S2. Design and Analytical Framework for Cost-Effectiveness Analysis of Childhood Myopia Control Strategies Based on a Markov Decision Model.

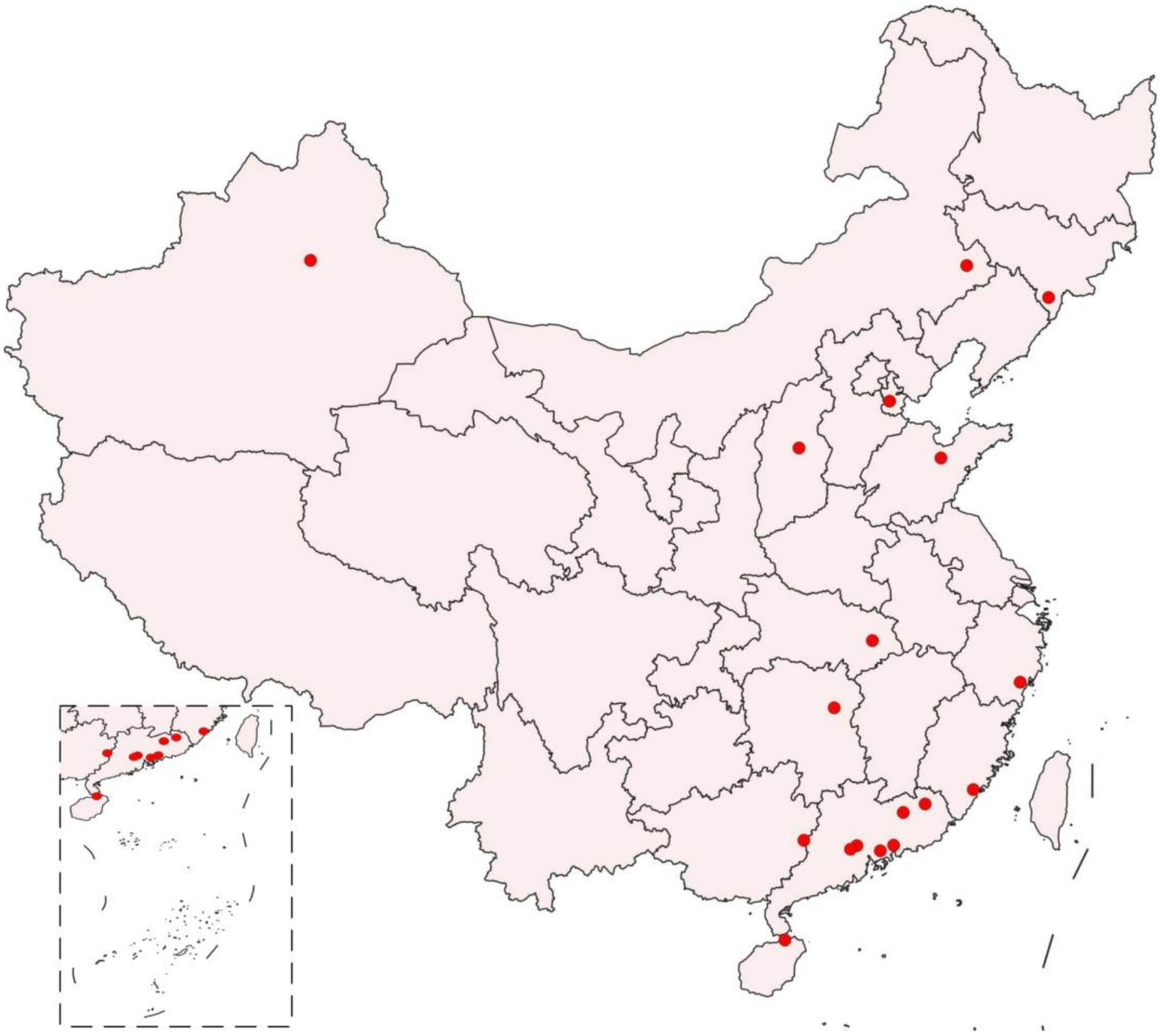

Figure S3. Distribution of sources for cost data in China**.**

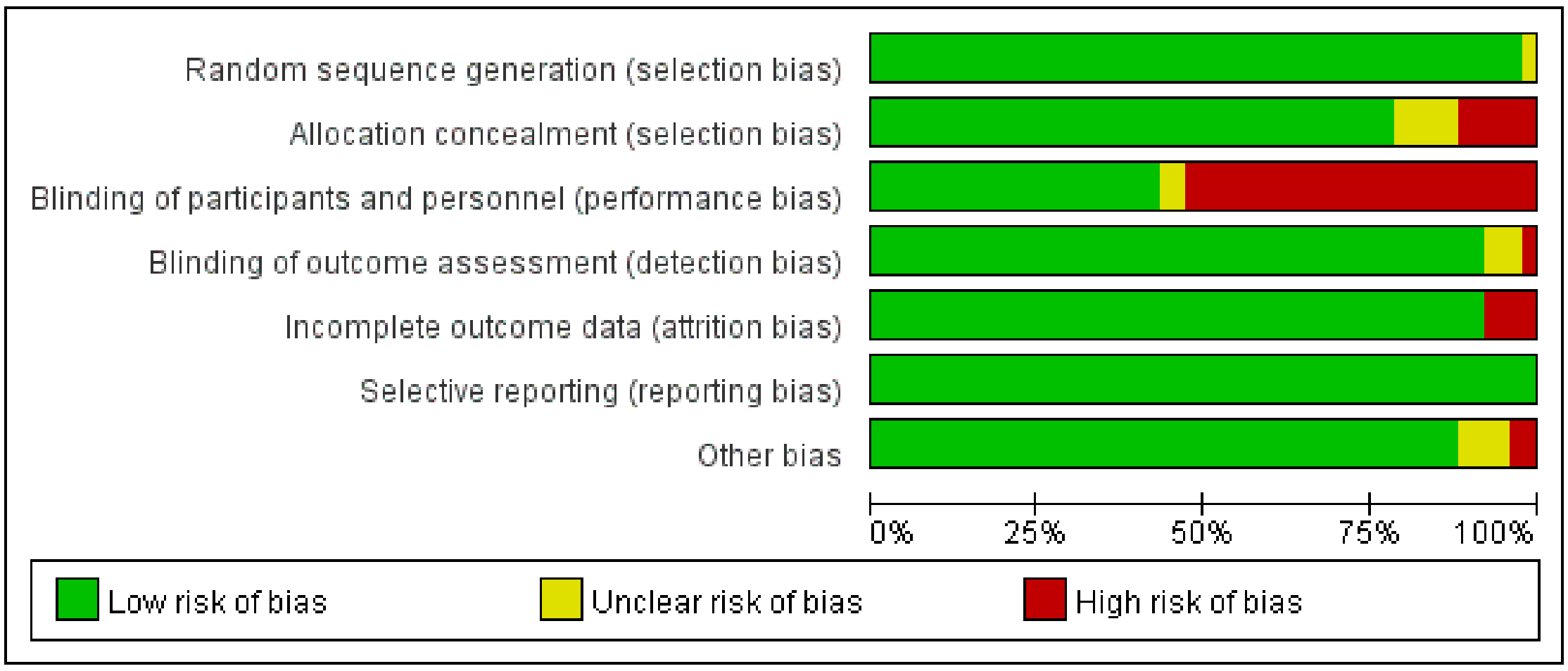

Figure S4. Risk of Bias Assessment (RCT).

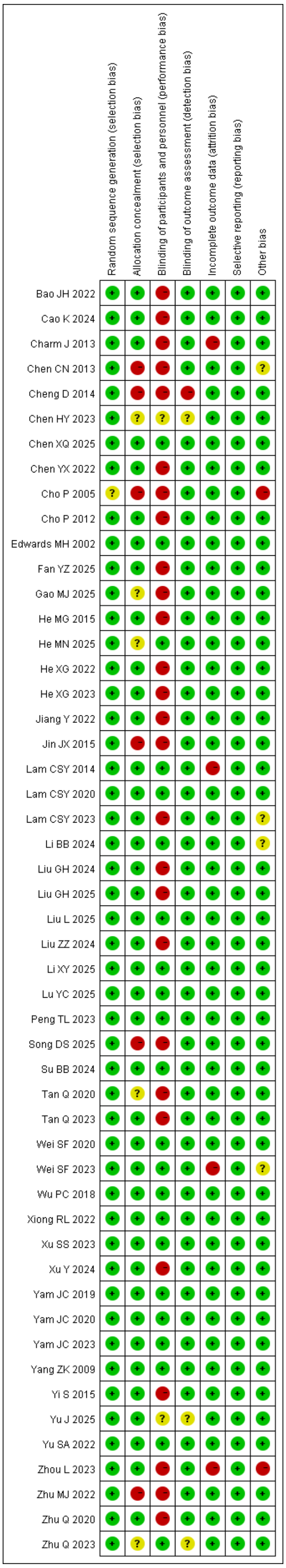

Figure S5. Summary of Risk of Bias in RCTs.

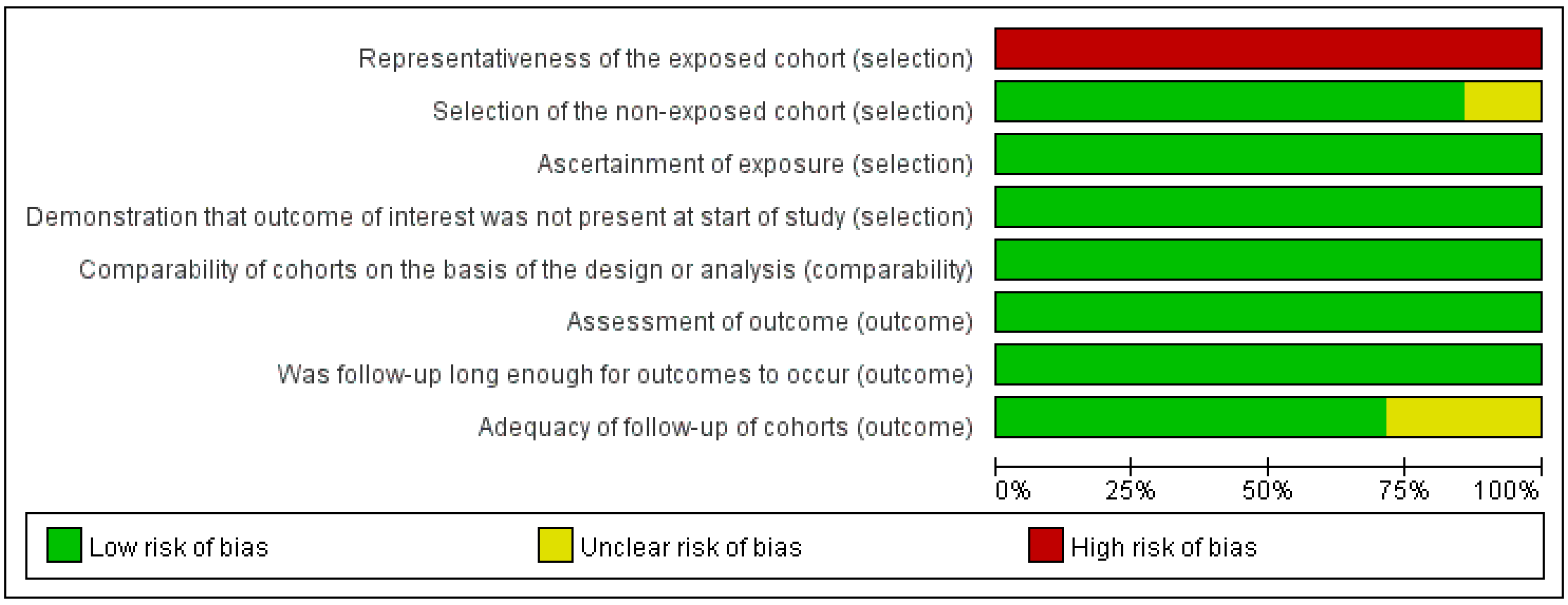

Figure S6. Risk of Bias Assessment (Cohort).

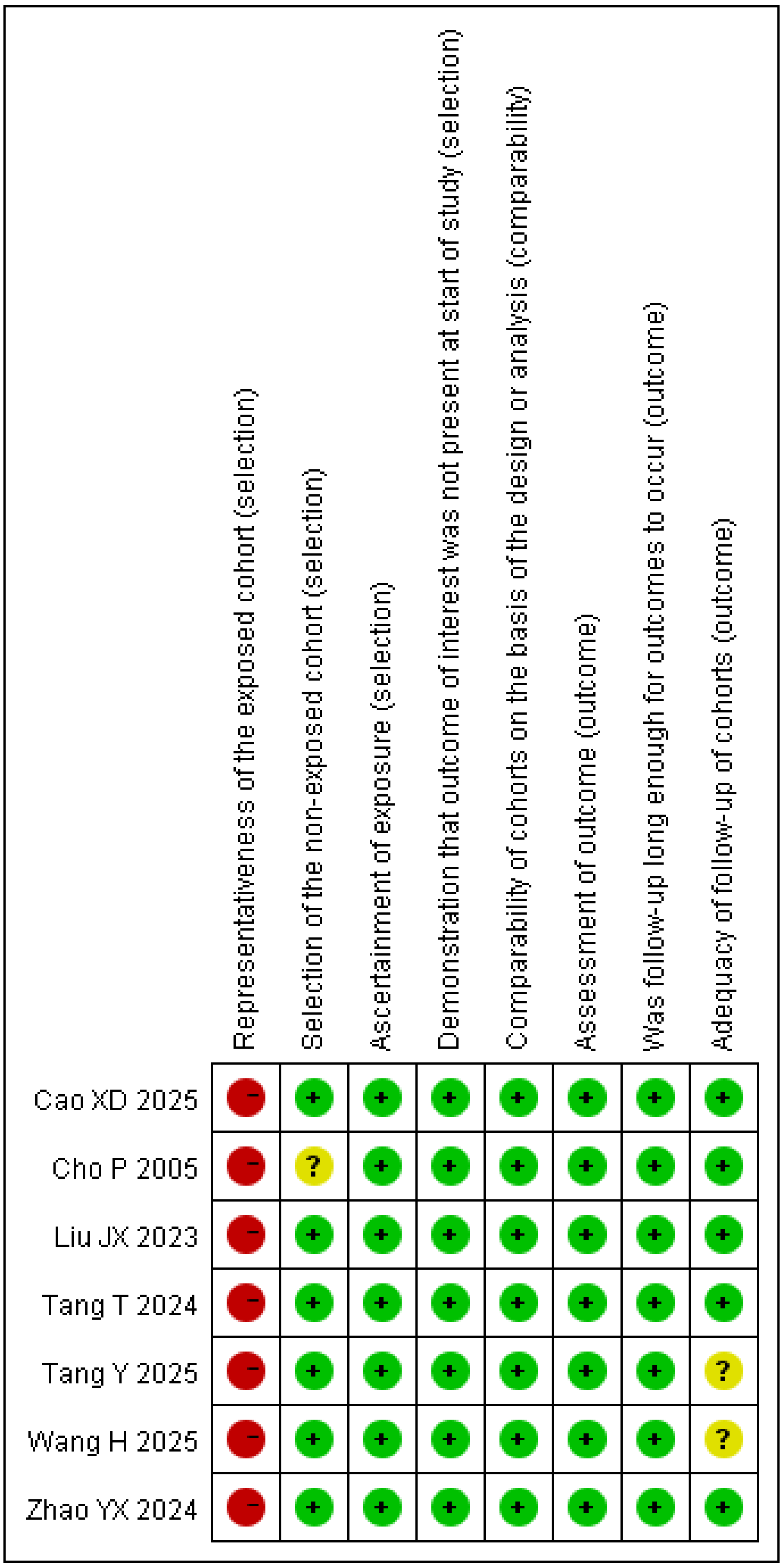

Figure S7. Summary of Risk of Bias in Cohorts.

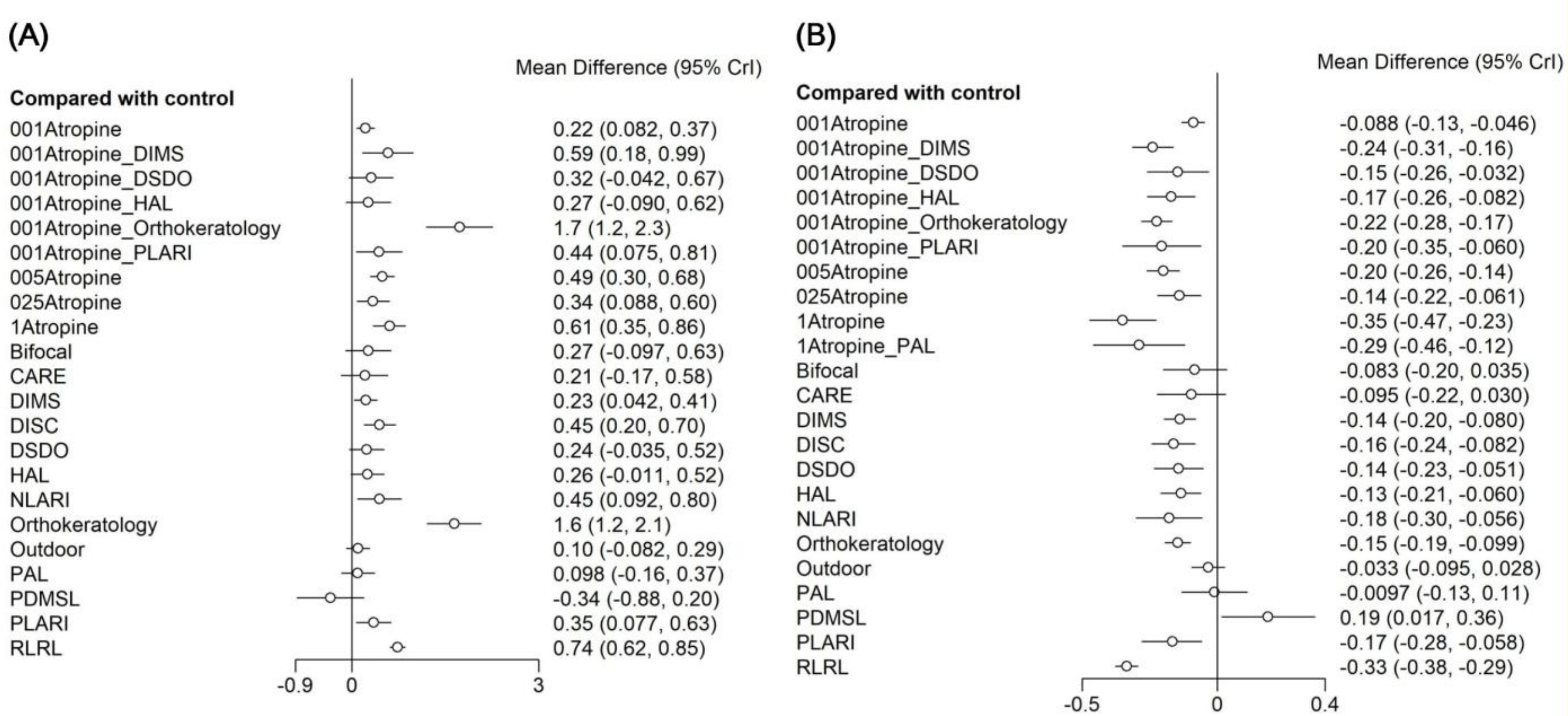

Figure S8. Forest plot for the control effects on SER and AL. Panel A. SER. Panel B. AL.

AL – axial length, Atr – atropine, CARE – cylindrical annular refractive element, DIMS – defocus incorporated multiple segment, DISC – defocus incorporated soft contact, DSDO – diversified segmental defocus optimisation, HAL – highly aspherical lenslet, HPLARI – lenslet-array-integrated spectacle lenses with positive power lenslets, NLARI – lenslet-array-integrated spectacle lenses with negative power lenslets, PAL – progressive addition lens, PDMSL – peripheral defocus modifying spectacle lens, RLRL – repeated low-level red-light therapy, SER – spherical equivalent refraction.

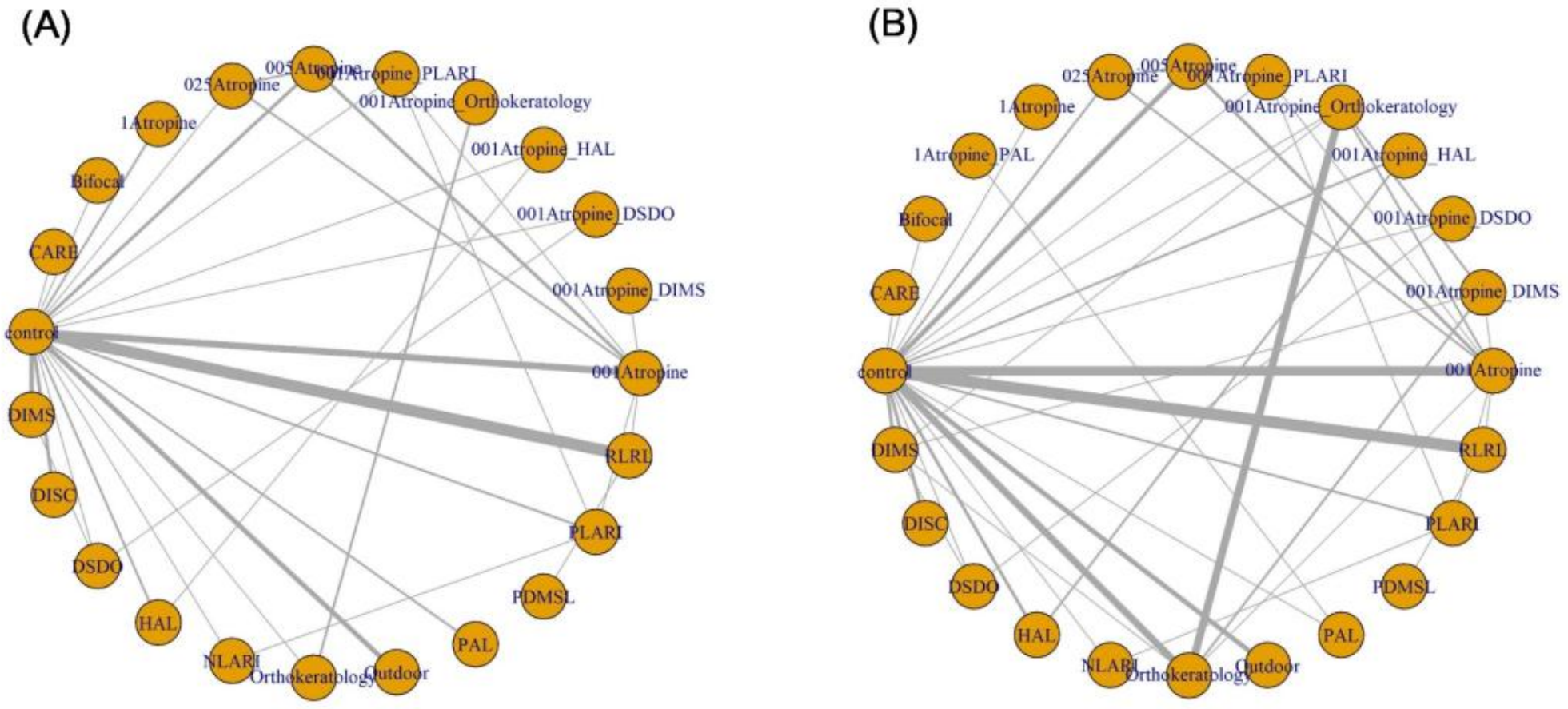

Figure S9. Network of myopia control interventions.

Note: (A) mean spherical refraction change; (B) mean axial length change.

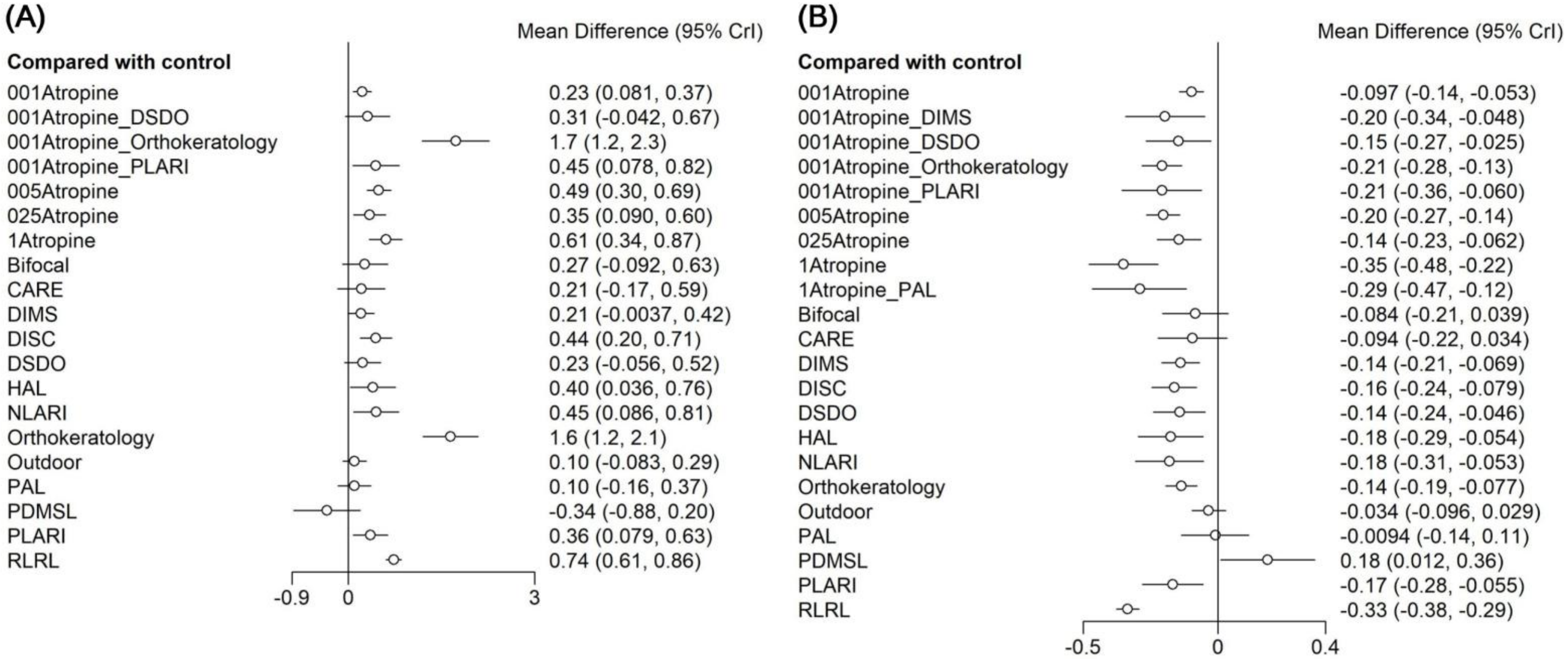

Figure S10. Forest plot for the control effects on SER and AL (including RCTs only). Panel A. SER. Panel B. AL.

AL – axial length, Atr – atropine, CARE – cylindrical annular refractive element, DIMS – defocus incorporated multiple segment, DISC – defocus incorporated soft contact, DSDO – diversified segmental defocus optimisation, HAL – highly aspherical lenslet, HPLARI – lenslet-array-integrated spectacle lenses with positive power lenslets, NLARI – lenslet-array-integrated spectacle lenses with negative power lenslets, PAL – progressive addition lens, PDMSL – peripheral defocus modifying spectacle lens, RLRL – repeated low-level red-light therapy, SER – spherical equivalent refraction.

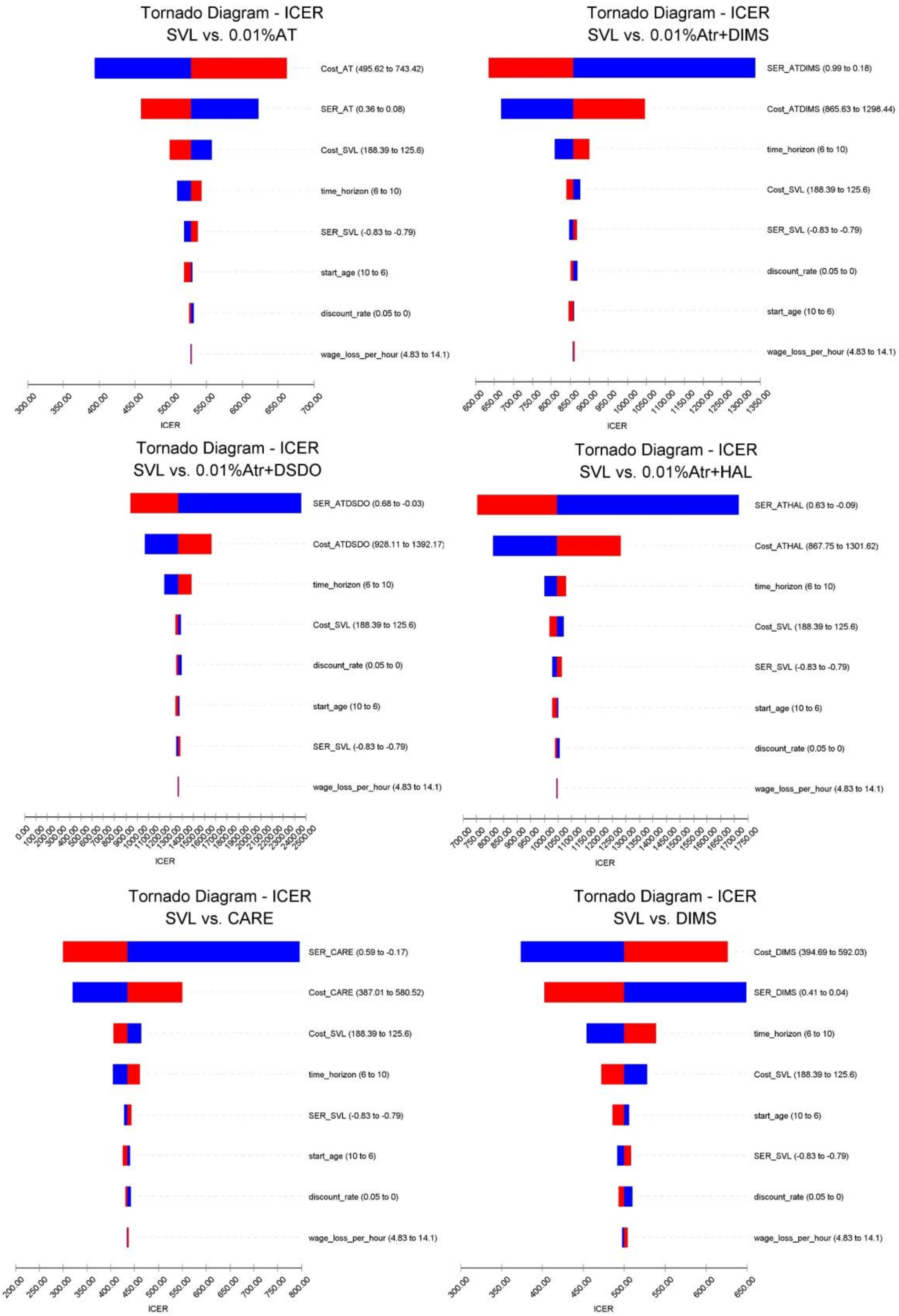

Figure S11. Tornado Diagram of Sensitivity Analysis on the Effect of SER.

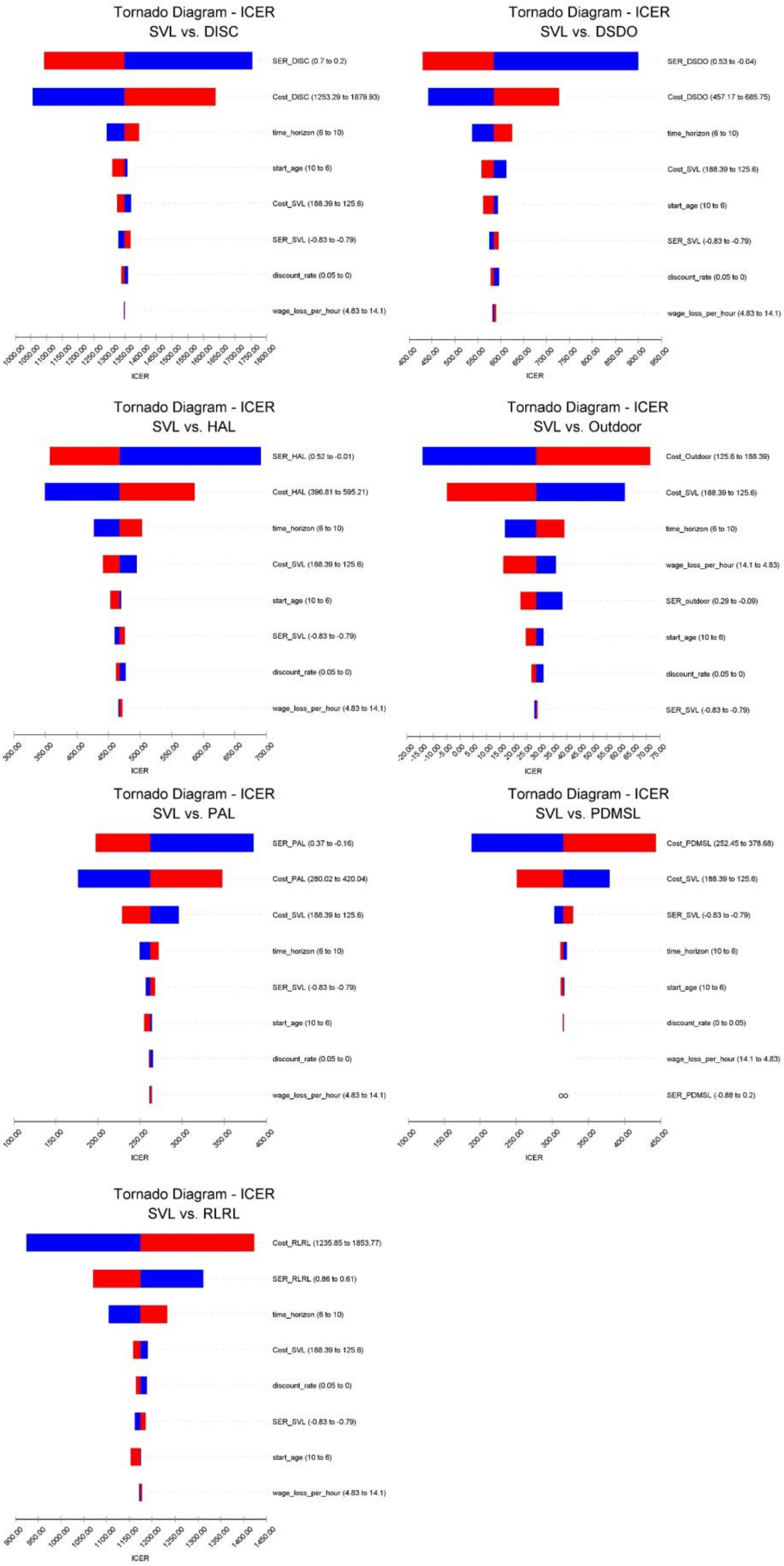

Figure S12. Tornado Diagram of Sensitivity Analysis on the Effect of SER.

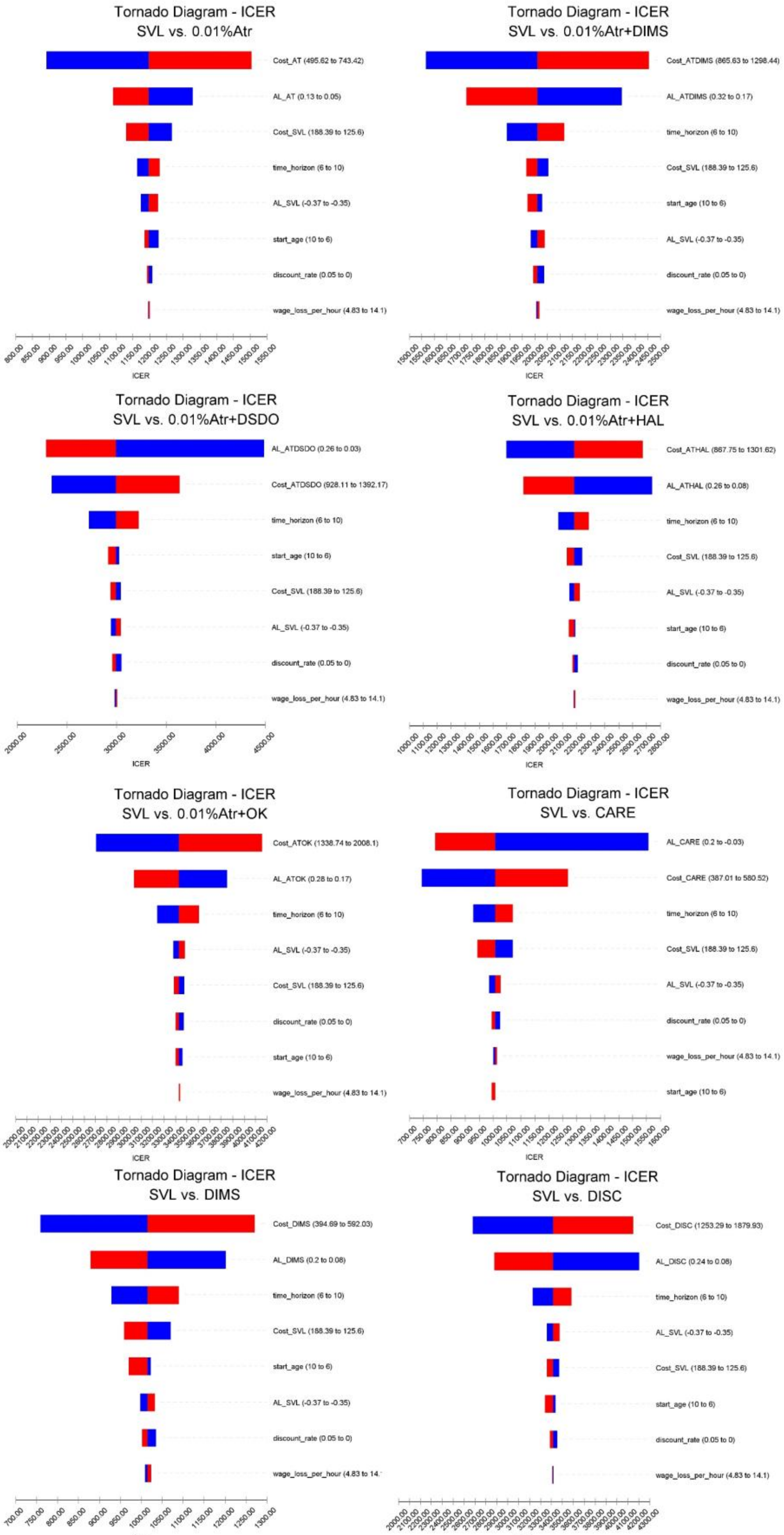

Figure S13. Tornado Diagram of Sensitivity Analysis on the Effect of AL.

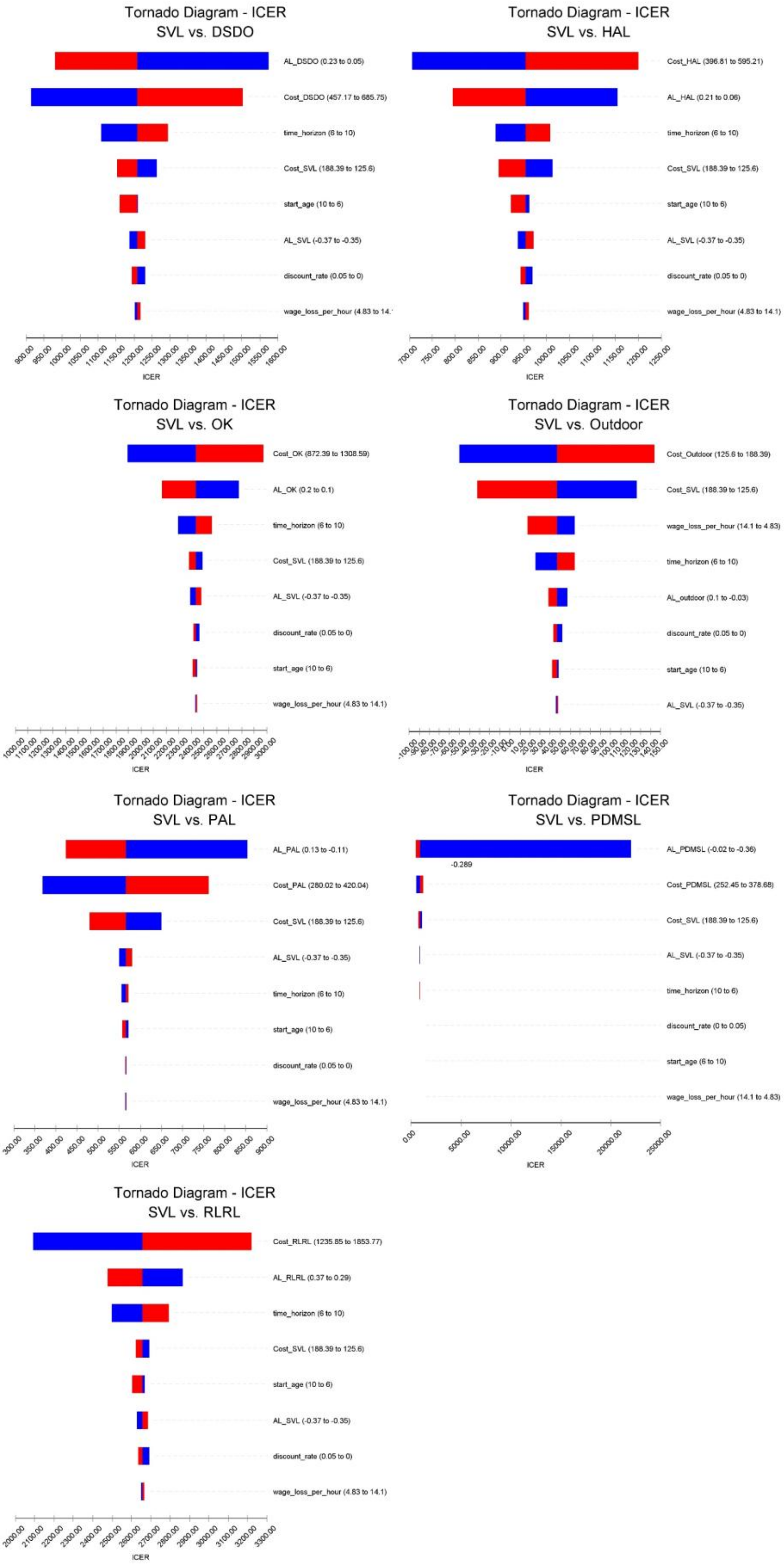

Figure S14. Tornado Diagram of Sensitivity Analysis on the Effect of AL.

References:

1. Bao J, Huang Y, Li X, et al. Spectacle Lenses With Aspherical Lenslets for Myopia Control vs Single-Vision Spectacle Lenses A Randomized Clinical Trial. *JAMA OPHTHALMOLOGY*. 2022;140(5):472-478. doi:10.1001/jamaophthalmol.2022.0401

2. Cao K, Tian L, Ma D, et al. Daily Low-Level Red Light for Spherical Equivalent Error and Axial Length in Children With Myopia. *JAMA OPHTHALMOLOGY*. 2024;142(6):560-567. doi:10.1001/jamaophthalmol.2024.0801

3. Chen H, Wang W, Liao Y, et al. Low-intensity red-light therapy in slowing myopic progression and the rebound effect after its cessation in Chinese children: a randomized controlled trial. *GRAEFES ARCHIVE FOR CLINICAL AND EXPERIMENTAL OPHTHALMOLOGY*. 2023;261(2):575-584. doi:10.1007/s00417-022-05794-4

4. Chen X, Wu M, Yu C, et al. Efficacy of Cylindrical Annular Refractive Elements (CARE) Spectacle Lenses in Slowing Myopia Progression Over 2 Years. *AMERICAN JOURNAL OF OPHTHALMOLOGY*. 2025;278:203-211. doi:10.1016/j.ajo.2025.06.017

5. Chen Y, Xiong R, Chen X, et al. Efficacy Comparison of Repeated Low-Level Red Light and Low-Dose Atropine for Myopia Control: A Randomized Controlled Trial. *Transl Vis Sci Technol*. 2022;11(10):33. doi:10.1167/tvst.11.10.33

6. Cheng D, Woo G, Drobe B, Schmid K. Effect of Bifocal and Prismatic Bifocal Spectacles on Myopia Progression in Children Three-Year Results of a Randomized Clinical Trial. *JAMA OPHTHALMOLOGY*. 2014;132(3):258-264. doi:10.1001/jamaophthalmol.2013.7623

7. Cho P, Cheung S, Edwards M. The longitudinal orthokeratology research in children (LORIC) in Hong Kong: A pilot study on refractive changes and myopic control. *CURRENT EYE RESEARCH*. 2005;30(1):71-80. doi:10.1080/02713680590907256

8. Edwards M, Li R, Lam C, Lew J, Yu B. The Hong Kong progressive lens myopia control study: Study design and main findings. *INVESTIGATIVE OPHTHALMOLOGY & VISUAL SCIENCE*. 2002;43(9):2852-2858.

9. Fan Y, Chu H, Peng Z, et al. Real-world outcomes on myopia management efficacy of diverse segmented defocus optics (DSDO) and defocus incorporated multiple segments (DIMS) spectacle lenses in Chinese children: An initial 12-month prospective clinical study. *J Optom*. 2025;18(1):100533. doi:10.1016/j.optom.2024.100533

10. Gao M, Hou Y, Lu Y, Shi Z, Zhao Q. Efficacy and Safety of 0.01% Atropine Eye Drops and Novel Lenslet-ARray-Integrated Spectacle Lenses for the Prevention of Myopia Progression Among Children with Premyopia: A Randomized Clinical Trial. *OPHTHALMOLOGY AND THERAPY*. 2025;14(10):2481-2496. doi:10.1007/s40123-025-01214-y

11. He M, Xiang F, Zeng Y, et al. Effect of Time Spent Outdoors at School on the Development of Myopia Among Children in China A Randomized Clinical Trial. *JAMA-JOURNAL OF THE*

AMERICAN MEDICAL ASSOCIATION. 2015;314(11):1142-1148. doi:10.1001/jama.2015.10803

12. He M, Wang H, Du B, et al. 0.01% Atropine for Myopia Prevention in Pre-myopia (AMPP study). *OPHTHALMIC AND PHYSIOLOGICAL OPTICS*. 2025;45(7):1924-1934. doi:10.1111/opo.70018

13. He X, Sankaridurg P, Wang J, et al. Time Outdoors in Reducing Myopia: A School-Based Cluster Randomized Trial with Objective Monitoring of Outdoor Time and Light Intensity. *Ophthalmology*. 2022;129(11):1245-1254. doi:10.1016/j.ophtha.2022.06.024

14. He X, Wang J, Zhu Z, et al. Effect of Repeated Low-level Red Light on Myopia Prevention Among Children in China With Premyopia: A Randomized Clinical Trial. *JAMA NETWORK OPEN*. 2023;6(4). doi:10.1001/jamanetworkopen.2023.9612

15. Jiang Y, Zhu Z, Tan X, et al. Effect of Repeated Low-Level Red-Light Therapy for Myopia Control in Children: A Multicenter Randomized Controlled Trial. *Ophthalmology*. 2022;129(5):509-519. doi:10.1016/j.ophtha.2021.11.023

16. Jin JX, Hua WJ, Jiang X, et al. Effect of outdoor activity on myopia onset and progression in school-aged children in northeast China: the Sujiatun Eye Care Study. *BMC Ophthalmol*. 2015;15:73. doi:10.1186/s12886-015-0052-9

17. Lam C, Tang W, Tse D, Tang Y, To C. Defocus Incorporated Soft Contact (DISC) lens slows myopia progression in Hong Kong Chinese schoolchildren: a 2-year randomised clinical trial. *BRITISH JOURNAL OF OPHTHALMOLOGY*. 2014;98(1):40-45. doi:10.1136/bjophthalmol-2013-303914

18. Lam C, Tang W, Tse D, et al. Defocus Incorporated Multiple Segments (DIMS) spectacle lenses slow myopia progression: a 2-year randomised clinical trial. *BRITISH JOURNAL OF OPHTHALMOLOGY*. 2020;104(3):363-368. doi:10.1136/bjophthalmol-2018-313739

19. Lam CSY, Tang WC, Zhang HY, et al. Long-term myopia control effect and safety in children wearing DIMS spectacle lenses for 6 years. *Sci Rep*. 2023;13(1):5475. doi:10.1038/s41598-023-32700-7

20. Li X, Ma W, Song Y, Yap M, Liu L. Comparison of Myopic Progression and Quality of Life Wearing Either DIMs Lenses or Single-Vision Myopia Correcting Spectacles. *JOURNAL OF OPHTHALMOLOGY*. 2025;2025(1). doi:10.1155/joph/9959251

21. Liu G, Rong H, Liu Y, et al. Effectiveness of repeated low-level red light in myopia prevention and myopia control. *Br J Ophthalmol*. 2024;108(9):1299-1305. doi:10.1136/bjo-2023-324260

22. Liu G, Liu L, Rong H, et al. Axial Shortening Effects of Repeated Low-level Red-light Therapy in Children With High Myopia: A Multicenter Randomized Controlled Trial. *Am J Ophthalmol*. 2025;270:203-215. doi:10.1016/j.ajo.2024.10.011

23. Liu J, Lu Y, Huang D, et al. The Efficacy of Defocus Incorporated Multiple Segments Lenses in Slowing Myopia Progression: Results from Diverse Clinical Circumstances. *Ophthalmology*. 2023;130(5):542-550. doi:10.1016/j.ophtha.2023.01.007

24. Liu L, Wang J, Tse DYY, et al. Effect of daily disposable Defocus Incorporated Soft Contact lens on myopia control: a 1-year multicentre randomised controlled trial. *Br J Ophthalmol*. Published online July 24, 2025:bjo-2025-327471. doi:10.1136/bjo-2025-327471

25. Liu Z, Sun Z, Du B, et al. The Effects of Repeated Low-Level Red-Light Therapy on the Structure and Vasculature of the Choroid and Retina in Children with Premyopia. *OPHTHALMOLOGY AND THERAPY*. 2024;13(3):739-759. doi:10.1007/s40123-023-00875-x

26. Lu Y, Yang X, Zhou J, et al. Diversified Segmental Defocus Optimization Lenses With and Without Atropine for Myopia Prevention A Randomized Clinical Trial. *JAMA OPHTHALMOLOGY*. 2025;143(8):684-691. doi:10.1001/jamaophthalmol.2025.2072

27. Peng T, Jiang J. Efficiency and Related Factors of Multifocal Soft Contact Lenses in Controlling Myopia. *EYE & CONTACT LENS-SCIENCE AND CLINICAL PRACTICE*. 2023;49(12):535-541. doi:10.1097/ICL.0000000000001043

28. Su B, Cho P, Vincent SJ, et al. Novel Lenslet-ARray-Integrated Spectacle Lenses for Myopia Control: A 1-Year Randomized, Double-Masked, Controlled Trial. *Ophthalmology*. 2024;131(12):1389-1397. doi:10.1016/j.ophtha.2024.07.002

29. Tan Q, Ng A, Choy B, Cheng G, Woo V, Cho P. One-year results of 0.01% atropine with orthokeratology (AOK) study: a randomised clinical trial. *OPHTHALMIC AND PHYSIOLOGICAL OPTICS*. 2020;40(5):557-566. doi:10.1111/opo.12722

30. Tan Q, Ng A, Cheng G, Woo V, Cho P. Combined 0.01% atropine with orthokeratology in childhood myopia control (AOK) study: A 2-year randomized clinical trial. *CONTACT LENS & ANTERIOR EYE*. 2023;46(1). doi:10.1016/j.clae.2022.101723

31. Wei S, Li S, An W, et al. Safety and Efficacy of Low-Dose Atropine Eyedrops for the Treatment of Myopia Progression in Chinese Children A Randomized Clinical Trial. *JAMA OPHTHALMOLOGY*. 2020;138(11):1178-1184. doi:10.1001/jamaophthalmol.2020.3820

32. Wei S, Li S, An W, et al. Myopia progression after cessation of low-dose atropine eyedrops treatment: A two-year randomized, double-masked, placebo-controlled, cross-over trial. *ACTA OPHTHALMOLOGICA*. 2023;101(2):E177-E184. doi:10.1111/aos.15235

33. Wu P, Chen C, Lin K, et al. Myopia Prevention and Outdoor Light Intensity in a School-Based Cluster Randomized Trial. *OPHTHALMOLOGY*. 2018;125(8):1239-1250. doi:10.1016/j.ophtha.2017.12.011

34. Xiong R, Zhu Z, Jiang Y, et al. Sustained and rebound effect of repeated low-level red-light therapy on myopia control: A 2-year post-trial follow-up study. *CLINICAL AND*

EXPERIMENTAL OPHTHALMOLOGY. 2022;50(9):1013-1024. doi:10.1111/ceo.14149

35. Xu Y, Cui L, Kong M, et al. Repeated Low-Level Red Light Therapy for Myopia Control in High Myopia Children and Adolescents: A Randomized Clinical Trial. *Ophthalmology*. 2024;131(11):1314-1323. doi:10.1016/j.ophtha.2024.05.023

36. Yam J, Jiang Y, Tang S, et al. Low-Concentration Atropine for Myopia Progression (LAMP) Study A Randomized, Double-Blinded, Placebo-Controlled Trial of 0.05%, 0.025%, and 0.01% Atropine Eye Drops in Myopia Control. *OPHTHALMOLOGY*. 2019;126(1):113-124. doi:10.1016/j.ophtha.2018.05.029

37. Yam J, Li F, Zhang X, et al. Two-Year Clinical Trial of the Low-Concentration Atropine for Myopia Progression (LAMP) Study Phase 2 Report. *OPHTHALMOLOGY*. 2020;127(7):910-919. doi:10.1016/j.ophtha.2019.12.011

38. Cao X, Guo Z, Wei Z, et al. Effect of 0.01% atropine eye drops combined with different optical treatments to control low myopia in Chinese children. *Cont Lens Anterior Eye*. 2025;48(1):102317. doi:10.1016/j.clae.2024.102317

39. Tang Y, Song J, Huang Y, Mao X. Comparison of the effects of spectacle lenses with highly aspherical lenslets combined with atropine for moderate to high myopia control in children. *BMC Ophthalmol*. 2025;25(1):700. doi:10.1186/s12886-025-04584-w

40. Yam J, Zhang X, Zhang Y, et al. Effect of Low-Concentration Atropine Eyedrops vs Placebo on Myopia Incidence in Children The LAMP2 Randomized Clinical Trial. *JAMA-JOURNAL OF THE AMERICAN MEDICAL ASSOCIATION*. 2023;329(6):472-481. doi:10.1001/jama.2022.24162

41. Yang Z, Lan W, Ge J, et al. The effectiveness of progressive addition lenses on the progression of myopia in Chinese children. *OPHTHALMIC AND PHYSIOLOGICAL OPTICS*. 2009;29(1):41-48. doi:10.1111/j.1475-1313.2008.00608.x

42. Yi S, Huang Y, Yu SZ, Chen XJ, Yi H, Zeng XL. Therapeutic effect of atropine 1% in children with low myopia. *J AAPOS*. 2015;19(5):426-429. doi:10.1016/j.jaapos.2015.04.006

43. Yu J, Li B, Li X, et al. Efficacy comparison between repeated low-level red-light therapy and peripheral defocus spectacles to slow the progression of medium to high myopia in Chinese children. *VISUAL NEUROSCIENCE*. 2025;42. doi:10.48130/vns-0025-0001

44. Zhou L, Tong L, Li Y, Williams BT, Qiu K. Photobiomodulation therapy retarded axial length growth in children with myopia: evidence from a 12-month randomized controlled trial evidence. *Sci Rep*. 2023;13(1):3321. doi:10.1038/s41598-023-30500-7

45. Zhu Q, Tang Y, Guo L, et al. Efficacy and Safety of 1% Atropine on Retardation of Moderate Myopia Progression in Chinese School Children. *INTERNATIONAL JOURNAL OF MEDICAL SCIENCES*. 2020;17(2):176-181. doi:10.7150/ijms.39365

46. Zhu Q, Tang G, Hua Z, et al. 0.05% atropine on control of myopia progression in Chinese school children: a randomized 3-year clinical trial. *INTERNATIONAL JOURNAL OF OPHTHALMOLOGY*. 2023;16(6):939-946. doi:10.18240/ijo.2023.06.17

47. Charm J, Cho P. High Myopia-Partial Reduction Ortho-k: A 2-Year Randomized Study. *OPTOMETRY AND VISION SCIENCE*. 2013;90(6):530-539. doi:10.1097/OPX.0b013e318293657d

48. Chen C, Cheung S, Cho P. Myopia Control Using Toric Orthokeratology (TO-SEE Study). *INVESTIGATIVE OPHTHALMOLOGY & VISUAL SCIENCE*. 2013;54(10):6510-6517. doi:10.1167/iovs.13-12527

49. Cho P, Cheung S. Retardation of Myopia in Orthokeratology (ROMIO) Study: A 2-Year Randomized Clinical Trial. *INVESTIGATIVE OPHTHALMOLOGY & VISUAL SCIENCE*. 2012;53(11):7077-7085. doi:10.1167/iovs.12-10565

50. Li B, Yu S, Gao S, et al. Effect of 0.01% atropine combined with orthokeratology lens on axial elongation: a 2-year randomized, double-masked, placebo-controlled, cross-over trial. *Front Med (Lausanne)*. 2024;11:1358046. doi:10.3389/fmed.2024.1358046

51. Song D, Chen Y, Yao J, Chen J. Effectiveness of DIMS combined with atropine and orthokeratology in a real-world setting in China. *CONTACT LENS & ANTERIOR EYE*. 2025;48(6). doi:10.1016/j.clae.2025.102473

52. Tang T, Lu Y, Li X, et al. Comparison of the long-term effects of atropine in combination with Orthokeratology and defocus incorporated multiple segment lenses for myopia control in Chinese children and adolescents. *Eye*. 2024;38(9):1660-1667. doi:10.1038/s41433-024-02987-5

53. Wang H, Piao J, Yoo G, Li D, Liu J, In-Chul J. Investigation into the Therapeutic Efficacy and Inflammatory Modulatory Effects of the Combined Use of 0.01% Atropine Eye Drops and Orthokeratology Lenses in the Management of Myopia Among Adolescent Patients. *J Inflamm Res*. 2025;18:12077-12087. doi:10.2147/JIR.S539831

54. Xu S, Li Z, Zhao W, et al. Effect of atropine, orthokeratology and combined treatments for myopia control: a 2-year stratified randomised clinical trial. *Br J Ophthalmol*. 2023;107(12):1812-1817. doi:10.1136/bjo-2022-321272

55. Yu S, Du L, Ji N, et al. Combination of orthokeratology lens with 0.01% atropine in slowing axial elongation in children with myopia: a randomized double-blinded clinical trial. *BMC Ophthalmol*. 2022;22(1):438. doi:10.1186/s12886-022-02635-0

56. Zhao Y, Yang B, Li X, Ma W, Liu L, Yan N. Efficacy of Combining Highly Aspherical Lenslets Spectacles With 0.01% Atropine Eye Drops in Myopia Control. *Sichuan Da Xue Xue Bao Yi Xue Ban*. 2024;55(5):1280-1287. doi:10.12182/20240960109

57. Zhu M, Ding L, Du L, et al. Photopic pupil size change in myopic orthokeratology and its

influence on axial length elongation. *INTERNATIONAL JOURNAL OF OPHTHALMOLOGY*. 2022;15(8):1322-1330. doi:10.18240/ijo.2022.08.15

58. Luo Y, Xie J, Liao W, et al. Improved control efficacy of a novel aspherical orthokeratology lens compared with the traditional three-zone spherical lens in a single-blind randomized clinical trial. *Cont Lens Anterior Eye*. 2025;48(4):102404. doi:10.1016/j.clae.2025.102404
